# Intensive care needs after hip and knee replacement: understanding risk profiles for severe postoperative complications

**DOI:** 10.1186/s10195-025-00862-x

**Published:** 2025-07-03

**Authors:** Dominik Emanuel Holzapfel, Tobias Kappenschneider, Sabrina Holzapfel, Marie Farina Schuster, Katrin Michalk, Patrick Auer, Timo Schwarz

**Affiliations:** 1 Department of Orthopaedic Surgery, Regensburg University Medical Center, Kaiser-Karl V.-Allee 3, 93077 Bad Abbach, Germany; 2 Department of Neonatology, University Children’s Hospital Regensburg, Hospital St. Hedwig of the Order of St John, University of Regensburg, Steinmetzstraße 1-3, 93049 Regensburg, Germany; 3 Department of Anaesthesiology, Regensburg University Medical Center, Asklepios Klinikum Bad Abbach, Kaiser-Karl V.-Allee 3, 93077 Bad Abbach, Germany

**Keywords:** Intensive care, ICU, Severe complications, Clavien–Dindo classification, TJA, TKA, THA, Arthroplasty, Frailty

## Abstract

**Background:**

The etiology of serious life-threatening events after total joint arthroplasty (TJA) is poorly elaborated and understood in literature. The purpose of this study was to identify independent predictors of postoperative intensive care following total hip arthroplasty (THA) and total knee arthroplasty (TKA) and to clarify the circumstances leading to these transfers.

**Material and methods:**

A total of 142 patients suffering from postoperative intensive care-dependent serious adverse events (Clavien–Dindo classification Grade IV, CD°IV) after THA or TKA were matched 1:1 with non-CD°IV patients using propensity score matching for age, sex, comorbidity (Charlson Comorbidity Index, CCI), and year of treatment. Possible predictive factors for the need of postoperative intensive care were initially evaluated using univariate tests, followed by multivariate regression analyses to identify independent predictors.

**Results:**

CD°IV transfers correlate with higher Hospitality Frailty Risk Score levels (HFRS) [mean 4.4 (standard deviation, SD 3.8) versus mean 3.0 (SD 3.0); *p* < 0.001], higher American Society of Anesthesiologists Physical Status Classification System (ASA) Scores [mean 2.5 (SD 0.6) versus mean 2.3 (SD 0.7); *p* = 0.02], a greater proportion of octogenarians [35.9% (*n* = 51) versus 23.9% (*n* = 34); *p* = 0.028] and a higher incidence of medical complications [97.9% (*n* = 139) versus 60.6% (*n* = 86); *p* < 0.001] compared with an adjusted control group after total joint arthroplasty (TJA).

Multivariate regression analysis confirmed “Frailty” (odds ratio, OR 1.14, 95% confidence intervals, CI 1.05–1.23, *p* = .002), preexisting cardiological (odds ratio, OR 2.0, 95% confidence intervals, CI 1.004–4.1, *p* = 0.049) and gastrointestinal secondary diagnoses (OR 3.0, 95% CI 1.3–6.9, *p* = 0.01), and intake of anticoagulants (OR 2.7, 95% CI 1.6–4.6, *p* < 0.001) as independent risk factors for CD°IV intensive care unit (ICU) transfers after TJA.

**Conclusions:**

Patients with CD°IV events after THA and TKA represent a complex, vulnerable, and multimorbid patient population. There is a need for a multidisciplinary approach that integrates prehabilitation and perioperative risk assessments to reduce the occurrence of severe, life-threatening events requiring ICU care.

**Level of evidence:**

Level III—retrospective cohort study.

*** Trial registration *:**

Retrospectively registered.

**Supplementary Information:**

The online version contains supplementary material available at 10.1186/s10195-025-00862-x.

## Introduction

Osteoarthritis (OA) significantly impacts the lives of a substantial proportion of adults worldwide [1]. This growing burden is largely driven by the demographic shift and an aging population [2], which have led to an increasing demand for surgical interventions such as TJA and revision procedures[3, 4]. Despite advancements in surgical techniques and perioperative care [5, 6], a small but notable subset of patients experiences severe complications requiring admission to intensive care units (ICU) after TJA. The implementation of FAST-Track and specialized orthogeriatric (SOG) concepts in TJA represents an initial step towards safely guiding vulnerable patient populations through the surgical process [7, 8]. Nonetheless, these unplanned admissions to the intensive care unit (ICU) following TJA occur in approximately 1–12% [9–12], representing a resource and cost-intensive aspect of postoperative care [13]. Despite the frequency of these severe events, the underlying risk profiles and mechanisms leading to ICU transfers remain inadequately understood, highlighting the need for further research.

The primary outcome of this study was to identify independent prognostic factors associated with life-threatening postoperative complications requiring ICU admission according to the Clavien–Dindo classification Grade IV (CD°IV) following total joint arthroplasty (TJA). Variables examined included the Hospital Frailty Risk Score (HFRS), the American Society of Anesthesiologists Physical Status Classification System (ASA), age, body mass index (BMI), type of arthroplasty (total hip or total knee arthroplasty), intrahospital medical and surgical complications, comorbidities, and the use of potentially high-risk medications.

The Clavien–Dindo classification system grades complications on a scale of I to V on the basis of the level of therapeutic intervention required, with CD°IV indicating life-threatening events that mandate intensive care management [14]. The secondary outcome was a detailed analysis of the most prevalent contributing factors among patients with CD°IV complications to better characterize the clinical profiles of high-risk patients and improve perioperative risk stratification.

## Materials and methods

### Study population and data collection

This study is a retrospective matched-pair analysis derived from a database, generated from the hospital information system and the department’s joint registry. All patients who underwent primary elective total hip arthroplasty (THA) or total knee arthroplasty (TKA) between 2010 and 2023 were included, forming a consecutive series. Only primary joint arthroplasties were included in this study. Cases involving preoperative fractures, significant deformities, revision procedures, or tumor endoprostheses were excluded from the analysis.

Finally, a total of 142 patients were identified in the in-house database using International Classification of Diseases, Tenth Revision (ICD-10) codes and Grade IV of the Clavien–Dindo classification (CD°IV) [14]. The Clavien–Dindo classification stratifies complications into five grades on the basis of the required therapeutic intervention, with Grade IV representing severe, life-threatening conditions requiring intensive care management [14]. This indicates that the patients were transferred to an external intensive care unit to undergo specialized medical management, encompassing continuous monitoring and therapeutic interventions. As our department of anesthesiology keeps annual statistics on our patient transfers requiring intensive care, it was possible to check all 142 patients for completeness of data in the above-mentioned period. A full dataset was available for all patients included in the analysis. For all CD°IV cases, a comprehensive review of both hospital discharge summaries and ICU-specific reports was conducted to confirm intensive care treatment and to determine the reasons for ICU admission. Diagnoses coded at the time of hospitalization and discharge were retrieved from the hospital information system (ORBIS^®^; Agfa Healthcare), including the corresponding ICD-10 codes. Coding was performed by professional clinical coders and subsequently verified by physicians using information from the patients’ medical records. Complications were assessed using ICD-10 codes documented at discharge. The Hospital Frailty Risk Score (HFRS) and Charlson Comorbidity Index (CCI) were calculated on the basis of ICD-10 codes recorded at admission [15, 16]. Additional available data from the clinical information system included age, sex, body mass index (BMI), operative procedure, associated comorbidities, medications, length of hospital stay, time of surgery in minutes, CD°IV cases, medical and surgical complications, and reoperations within 90 days.

Propensity score matching (1:1) was conducted on the basis of age, sex, and comorbidity (Charlson Comorbidity Index, CCI) and year of treatment. The final dataset consisted of 284 patients: 142 requiring intensive care (CD°IV) and 142 matched non-CD°IV controls. Baseline characteristics were extracted from patient records.

### Endpoints

The primary endpoint was the identification of CD°IV predictors. Additional data collected included associated comorbidities, revision surgeries within 90 days, and postoperative complications. Comorbidities and preoperative parameters recorded included cardiological, pulmonary, renal, gastrointestinal, neurological, oncological, and rheumatological diseases, as well as depression, diabetes, osteoporosis, preoperative anemia, malnutrition, nicotine abuse, chronically elevated CRP levels, and medications such as disease-modifying antirheumatic drugs (DMARDs), corticosteroids, platelet aggregation inhibitors (aspirin or P2Y12 antagonists), coumarin derivatives, or immunosuppressive agents. Perioperative complications were categorized into surgical complications (e.g., periprosthetic joint infection, periprosthetic fracture, wound healing disorders, hematoma, aseptic loosening and subsidence, dislocation, instability, wear of mobile components, arthrofibrosis, range-of-motion limitation, anterior knee pain, tendon rupture, and nerve injuries) and medical complications (e.g., cardiological, pulmonary, renal, thromboembolic, neurological, and cerebrovascular AEs; delirium; urinary tract infection; electrolyte imbalances; and anemia requiring transfusion). Complications were defined and assessed on the basis of ICD-10 codes documented at discharge.

The secondary endpoint consisted of a detailed analysis of the primary causes leading to intensive care admissions among these patients.

### Surgical techniques

All procedures were performed within a single Department of Orthopedic Surgery at a University Medical Center. Patients received standardized treatment protocols for THA and TKA, including a unified rehabilitation program. The majority of patients underwent spinal anesthesia, with a minority receiving general or regional anesthesia. THA was performed in the lateral decubitus position using a minimally invasive anterolateral approach with cementless or cemented fixation. TKA procedures employed a cemented technique via a medial parapatellar approach without patellar resurfacing.

### Statistics

Propensity score matching (PSM) was utilized to balance baseline characteristics between the CD°IV and non-CD°IV groups including age, sex, Charlson Comorbidity Index (CCI), and year of treatment. A nearest-neighbor algorithm with a caliper width of 0.012 was used, chosen to ensure a high level of comparability between matched cases. Cases outside the region of common support were excluded. PSM and statistical analysis were conducted using IBM SPSS Statistics (version 29.0.0) with a significance level set at *p* ≤ 0.05. Continuous variables are presented as means ± standard deviation (SD) and medians with interquartile ranges (IQR). Categorical data are reported as absolute (*n*) and percentage (%) frequencies. Continuous variables were tested for normal distribution using the Shapiro–Wilk test; as all variables were nonnormally distributed, nonparametric methods such as the Mann–Whitney *U* test were applied for independent samples. Categorical variables were analyzed using the chi-squared test, and Fisher’s exact test was applied for small sample sizes or low expected frequencies. Multivariate logistic regression models were employed to identify independent predictors of postoperative CD°IV cases. Separate models were developed for preoperative and perioperative covariates, as well as for knee and hip arthroplasty, to account for the differing clinical and procedural factors influencing outcomes in these contexts. A forward selection procedure was applied, including variables with a univariate *p*-value < 0.10 in the model development process. These analyses facilitated the identification of confounders and independent predictors of CD°IV complications.

## Results

### Baseline characteristics

Between January 2010 and December 2023, a total of 34,455 patients were identified through the local hospital computer database. Among these, 14,505 cases underwent primary elective THA or TKA. Within this cohort, 142 patients (0.98%) suffered from severe adverse events requiring ICU admission, classified as CD°IV. One patient had a deadly event and passed away after unsuccessful cardiopulmonary resuscitation.

The baseline characteristics of the CD°IV group and the control group, including age, sex, and Charlson Comorbidity Index (CCI), were well balanced through propensity score matching. Baseline characteristics are summarized in Table 1.

**Table 1:** Baseline characteristics TJA

Total joint arthroplasty (TJA)				
	Controls (*n* = 142)	CD°IV patients (*n* = 142)	Total population (*n* = 284)	*p*-Value
**♀** *n* (%)	98 (69.0)	94 (66.2)	192 (67.6)	0.612*
**♂** *n* (%)	44 (31.0)	48 (33.8)	92 (32.4)	0.612*
**Age (years)** Mean (SD) Median (IQR)	73.4 (8.6) 74.0 (10.0)	75.1 (8.8) 77.0 (13.0)	74.2 (8.8) 76.0 (11)	0.064**
**CCI** Mean (SD) Median (IQR)	3.6 (1.7) 3.0 (1.0)	3.8 (1.5) 4.0 (1.0)	3.7 (1.6) 3.5 (1.0)	0.179**
**THA** *n* (%)	66 (46.5)	78 (54.9)	144 (50.7)	0.154*
**TKA** *n* (%)	76 (53.5)	64 (45.1)	140 (49.3)	0.154*
**Side left** *n* (%)	56 (39.4)	67 (47.2)	123 (43.3)	0.120**
**Side right** *n* (%) **Side both** *n* (%)	86 (60.6) 0 (0)	73 (51.4) 2 (1.4)	159 (56.0) 2 (0.7)	
**BMI** Mean (SD) Median (IQR) **Adipositas (yes/no)** *n* (%) **Adipositas ≥ II** *n* (%)	29.6 (6.7) 28.8 (9.3) 64 (63.4) 24 (39.3)	29.1 (6.1) 27.9 (7.8) 58 (60.4) 27 (41.5)	29.3 (6.4) 28.5 (8.7) 122 (61.9) 51 (40.5)	0.541** 0.670* 0.802*
**HFRS** Mean (SD) Median (IQR)	3.0 (3.0) 2.3 (2.3)	4.4 (3.8) 3.2 (4.8)	3.7 (3.5) 2.3 (3.4)	< 0.001**
**ASA score** Mean (SD) Median (IQR)	2.3 (0.7) 2.0 (1.0)	2.5 (0.6) 3.0 (1.0)	2.4 (0.6) 2.0 (1.0)	0.020**
**LOS (days)** Mean (SD) Median (IQR)	9.4 (3.2) 9.0 (3.0)	4.9 (4.5) 4.0 (4.0)	7.2 (4.5) 7.0 (5.0)	< 0.001**
**Time of surgery (minutes)** Mean (SD) Median (IQR)	75.5 (21.6) 74.0 (29.0)	78.1 (25.0) 73.0 (30.3)	76.8 (23.4) 73.5 (29.5)	0.594**

Clavien–Dindo IV (CD°IV): life-threatening complications leading to transfer to an intermediate care unit or intensive care unit. Age in years. Adipositas (yes/no) = BMI ≥ 30 versus normal weight patients; Adipositas ≥ II = BMI ≥ 35 versus normal weight patients. Time of surgery in minutes, bold values = *p* ≤ 0.05; *chi-squared test. **Mann–Whitney *U* test *CCI* Charlson Comorbidity Index, *TJA* total joint arthroplasty, *THA* total hip arthroplasty, *TKA* total knee arthroplasty, *BMI *body mass index, *ASA* American Society of Anesthesiologists, *LOS* length of stay in days

Bold values = p ≤ 0.05

### Primary outcome: influencing factors on CD°IV transfers

#### “Frailty”

**Fig. 1:**
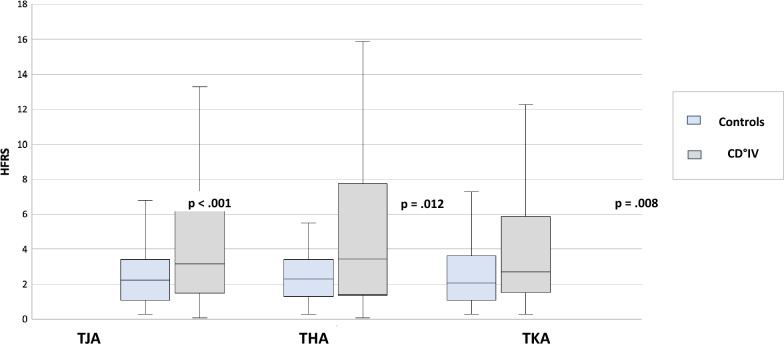
HFRS values in CD°IV patients versus control group after TJA, THA, and TKA. *HFRS* Hospitality Frailty Risk Score, *THA* total hip arthroplasty, *TKA* total knee arthroplasty, *TJA* total joint arthroplasty, *CD°IV* severe life-threatening complications requiring ICU transfer according to Clavien–Dindo classification Grade IV

The CD°IV cohort across all TJA cases demonstrated a significantly higher proportion of “Frailty” based on the HFRS compared with the control group, with a mean score of 4.4 (SD 3.8) versus 3.0 (SD 3.0) (*p* < 0.001). These findings were also confirmed for both THA [mean 4.9 (SD 4.4) versus mean 3.3 (SD 3.5); *p* = 0.012] and TKA [mean 3.7 (SD 2.6) versus mean 2.8 (SD 2.5); *p* = 0.008]. Figure 1 shows a graphical representation.

In the multivariate analysis, the HFRS was confirmed as an independent influencing factor for a transfer to CD°IV after all TJAs (OR 1.14, 95% CI 1.05–1.23, *p* = 0.002) and after THA (OR 1.12, 95% CI 1.01–1.23, *p* = 0.024).

#### Octogenarians (age ≥ 80 years)

A closer look at the group of over 80-year-old patients revealed a higher rate of CD°IV transfers than in the control group after TJA [35.9% (*n* = 51) versus 23.9% (*n* = 34); *p* = 0.028]. This also applies to the TKA group [40.6% (*n* = 26) versus 23.7% (*n* = 18); *p* = 0.031].

#### ASA-score

The analysis of the ASA score indicated a poorer preoperative physical health status among CD°IV patients compared with the control group in all TJA patients [mean 2.5 (SD 0.6) versus mean 2.3 (SD 0.7); *p* = 0.02] and THA patients [mean 2.6 (SD 0.5) versus mean 2.3 (SD 0.7); *p* = 0.004].

#### Complications

##### Surgical complications

There was no association between CD°IV transfer and pooled surgical complications. In detail, postoperative hematoma was the only surgical factor associated with an increased incidence of CD°IV transfers for all TJA [12.0% (*n* = 17) versus 3.5% (*n* = 5); *p* = 0.008] as well as for THA [12.8% (*n* = 10) versus 3.0% (*n* = 2); *p* = 0.034].

##### Medical complications

**Fig. 2:**
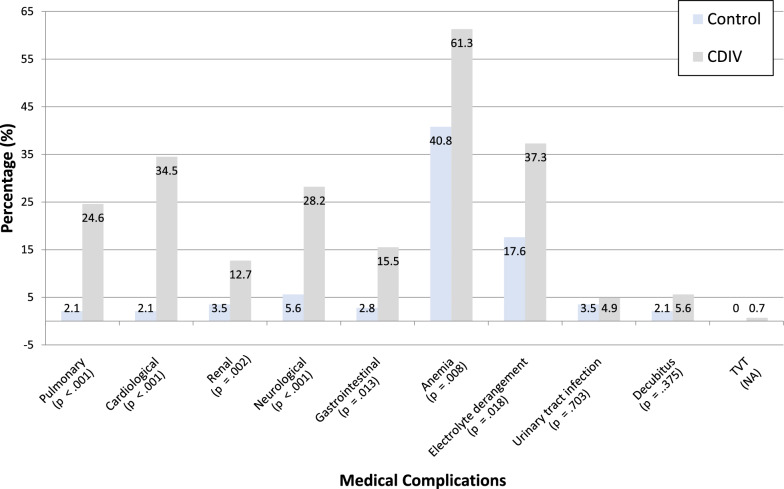
Medical complications after TJA. Clavien–Dindo IV (CD°IV) refers to life-threatening complications leading to transfer to an intermediate care unit or intensive care unit *TJA* total joint arthroplasty

The pooled medical complications demonstrated significantly higher rates of ICU transfers according to CD°IV compared with the control group in the univariate analysis after TJA [97.9% (*n* = 139) versus 60.6% (*n* = 86); *p* < 0.001] but also after THA [100% (*n* = 78) versus 66.7% (*n* = 44); *p* < 0.001] and TKA [95.3% (*n* = 61) versus 55.3% (*n* = 42); *p* < 0.001]. The leading causes of these medical complications after TJA are comprehensively detailed in Fig. 2 and Appendix S1 for further evaluation.

In the multivariate analyses, independent medical predictors for CD°IV ICU transfers following TJA were identified. Independent predictors were pulmonary complications (OR 42.2, 95% CI 11.0–161.6, *p* < 0.001), cardiological complications (OR 101.9, 95% CI 27.4–379.2, *p* < 0.001), renal complications (OR 15.6, 95% CI 4.7–51.9, *p* < 0.001), neurological complications (OR 20.1, 95% CI 7.5–53.6, *p* < 0.001), gastrointestinal complications (OR 31.4, 95% CI 9.1–107.8, *p* < 0.001) and postoperative anemia (OR 2.1, 95% CI 1.0–4.3, *p* = 0.047).

A detailed presentation of the multivariate analysis can be found in Appendix S2.

#### Comorbidities and potentially risk generating medication

##### Secondary diagnoses

In univariate analysis, patients with CD°IV transfers exhibited significantly higher rates of cardiological (88.0% versus 76.1%, *p* = 0.009), neurovascular (29.6% versus 17.6%, *p* = 0.018), hemato‐oncological (23.2% versus 14.1%, *p* = 0.048), and gastrointestinal comorbidities (17.6% versus 6.3%, *p* = 0.003) compared to controls.

##### Potentially risk generating medication:

Anticoagulant use was also significantly elevated in CD°IV patients (48.6% versus 23.9%, *p* < 0.001).

Appendix S3 and S4 provide an overview of the secondary diagnoses and the potentially risk-generating medication that correlate significantly with an ICU transfer after CD°IV.

The multivariate calculations of the above-mentioned influencing variables revealed the following independent predictors for CD°IV ICU transfers.

##### Secondary diagnoses:

Independent predictors of CD°IV after TJA were cardiological concomitant diseases (OR 2.0, 95% CI 1.004–4.1, *p* = 0.049) and gastrointestinal comorbidities (OR 3.0, 95% CI 1.3–6.9, *p* = 0.01). A detailed presentation of the multivariate analysis can be found in Appendix S5.

##### Potentially risk generating medication

Pooled anticoagulant intake was an independent predictor for CD°IV transfers after TJA (OR 2.7, 95% CI 1.6–4.6, *p* < 0.001), THA (OR 2.3, 95% CI 1.1–4.8, *p* = 0.023), and TKA (OR 4.1, 95% CI 1.9–8.6, *p* < 0.001).

**Fig. 3:**
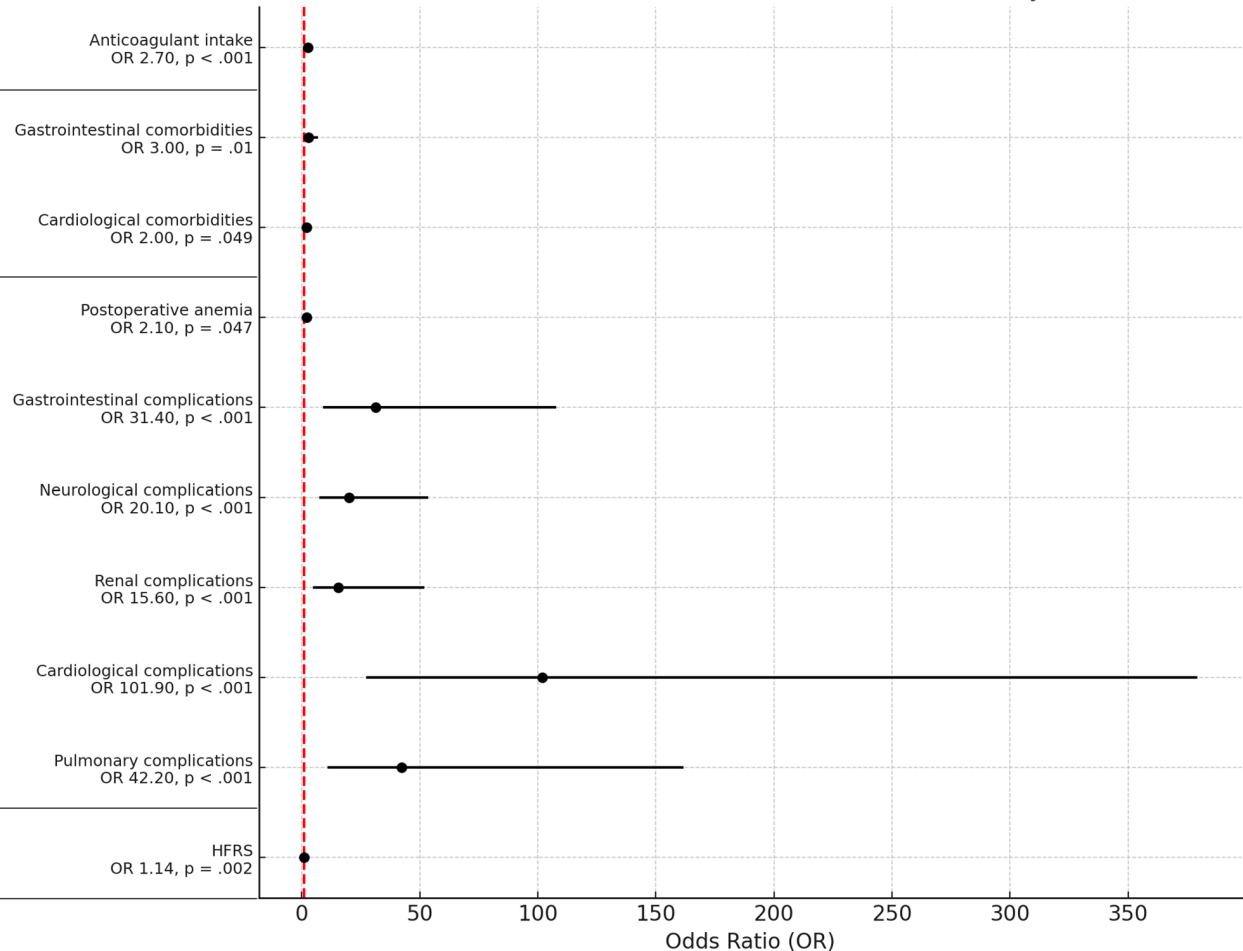
Independent predictors for CD°IV transfers after TJA. Clavien–Dindo IV (CD°IV)refers to life-threatening complications leading to transfer to an intermediate care unit or intensive care unit. Red dashed line = marks an OR of 1. *TJA* total joint arthroplasty, *HFRS* Hospitality Frailty Risk Score, *OR* odds ratio

Figure 3 shows a visual summary of the most important significant results of all multivariate logistic regression models according to TJA graphically presented in a forest plot.

##### Secondary outcome: analysis of the underlying causes for CD°IV transfers necessitating intensive care

The secondary outcome focused on identifying the key drivers of CD°IV transfers requiring intensive care. Cardiological events represent the primary cause of CD°IV transfers, comprising approximately one third of all cases. The most common cardiological reasons, in descending order, include acute myocardial infarction, acute coronary syndromes, arrhythmias (e.g., tachyarrhythmia absoluta, newly developed atrial fibrillation, etc.), cardiac decompensation, and hypertensive crisis.

Neurovascular events and pulmonary complications follow as the second and third most frequent reasons, respectively. Neurological serious adverse events (SAEs) include transient ischemic attacks (TIA)/strokes, exacerbated severe delirium, severe epileptic seizures, and other neurological causes. Pulmonary SAEs mainly consist of severe pneumonia, pulmonary embolism, and severe respiratory decompensation.

The fourth most common cause of CD° IV transfers are gastrointestinal events. These include symptoms of an acute abdomen, such as (sub)ileus events, cholecystolithiasis requiring endoscopic retrograde cholangiopancreatography (ERCP), incarceration due to recurrent incisional hernia, upper gastrointestinal bleeding, severe gastritis, and one case of pancolitis related to a severe *Clostridium difficile* infection.

Approximately 10% of cases are attributed to hemorrhagic events, consisting of severe hemorrhages requiring vascular surgical intervention and hemorrhagic shock resulting from acute bleeding.

**Fig. 4:**
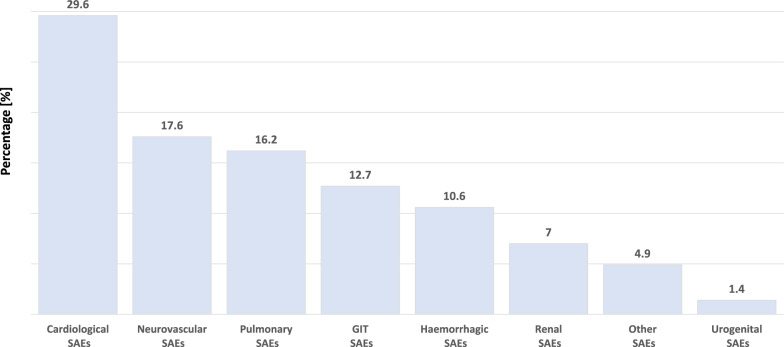
Main causes for CD°IV transfers to an ICU. *n* = 142 CD°IV transfers. *SAE* serious adverse event, *GIT* gastrointestinal

Urogenital events and “other” causes account for a minor proportion of cases. “Other” events include single cases such as severe opioid intoxication with respiratory insufficiency, severe drug intoxication resulting in transient tetraparesis, agranulocytosis following metamizole use, severe drug-induced hyponatremia, severe blood pressure dysregulation associated with pheochromocytoma, severe sepsis with multiorgan failure (renal insufficiency requiring dialysis, myocardial infarction, and severe pneumonia), and severe coronavirus disease 2019 (COVID-19) infection during the pandemic. Figure 4 shows the most common reasons for CD°IV relocations.

## Discussion

After the matching process, the groups exhibited a well-balanced and comparable distribution in terms of age, sex, BMI, type of arthroplasty (THA or TKA), and time of surgery in minutes. The patient population was characterized by overweight tendencies, with an average BMI of 29.3 (classified as preobesity), a relatively advanced mean age of 74 years, and a substantial burden of comorbidities, reflected in an average CCI of 3.7. Due to the ICU transfers, CD°IV patients exhibited a shorter average hospital stay, at 4.9 days. The overall mean LOS across the cohort was 7.2 days, showing a more accurate reflection of routine clinical practice in Germany [17]. The overall cohort consisted predominantly of female patients, with a ratio of approximately 2:1 in CD°IV patients compared with the control group. Courtney et al. also reported higher rates of ICU admissions among women in their research. However, literature does not support this finding as an independent predictor [18]. With a rate of approximately 1% CD°IV transfers in our patient cohort, this percentage falls at the lower end of the range of 1–12% [9–12] reported in literature. This also offers a realistic representation of routine clinical practice.

Meyer et al. (2020) demonstrated in retrospective analysis that a high HFRS constitutes an independent risk factor for adverse outcomes following TJA, including reoperations (OR 2.1, 95% CI 1.46–3.09, *p* < 0.001) and complications (internal complications: OR 3.72; 95% CI 2.28–6.08, *p* < 0.001; surgical complications: OR = 3.74, 95% CI 2.41–5.82, *p* < 0.001) [19]. Extending these findings, our analysis identified the HFRS as an independent predictor of CD°IV ICU transfers following TJA (OR 1.14; 95% CI 1.05–1.23; *p* = 0.002). While the endpoints differ, our results contribute novel insights to the current body of evidence and underscore the clinical relevance of frailty assessment in this context.

In the field of orthogeriatrics, significant advancements have been made in research over the past years [20, 21]. Specialized orthogeriatric concepts focus on prehabilitation, geriatric internal medicine, and intensive physiotherapeutic care in combination with FAST-Track concepts to improve postoperative outcomes following TJA. Multiple studies have shown that “frailty” is an independent predictor for postoperative complications after primary TJA [19, 22–24]. The results of the present study confirmed this finding for severe intensive-care complications classified as CD°IV. Additionally, the age group of octogenarians was identified as a possible predictor for these transfers. These findings add valuable insights to existing literature, particularly by highlighting the role of “frailty” and advanced age as important predictors in orthogeriatric outcomes.

Focusing on the ASA score, previous studies have demonstrated that patients preoperatively classified as ASA 3 and 4 experience significantly higher rates of intraoperative and postoperative complications, as well as prolonged intensive care unit (ICU) stays. Our findings corroborate these results [25].

Postoperative complications showed distinct patterns between surgical and medical events. Our findings revealed no increased incidence of surgical complications within the CD°IV transfer group. We hypothesize that while surgical complications may contribute to adverse outcomes, their severity in our cohort did not necessitate ICU transfers. Specifically, only postoperative hematomas were significantly correlated with CD°IV transfers. This may also be associated with the use of anticoagulants and postoperative anemia, as both emerged as independent predictors of CD°IV transfers for all TJAs, consistent with current literature [9].

A closer examination of the medical complications indicates a significant association with CD°IV transfers. In particular, pulmonary, cardiological, renal, neurological, and gastrointestinal complications were identified as independent predictors of CD°IV transfers. The wide confidence intervals and extremely high odds ratio for cardiological complications reflect a limited number of events and high variability in this subgroup. Although the association remained statistically significant, the magnitude of the effect should be interpreted with caution. These results supports the outcome of other studies and expands the scope of results [26–28]. Although both medical and surgical complications appear to be relevant components contributing to ICU transfers, further clinical research is necessary to differentiate their specific impact and to develop targeted strategies for early identification and risk mitigation. In clinical reality, ICU transfers are often triggered by a cumulative burden of complications rather than a single isolated event. Future research should aim to model these interactions more precisely. Given their potential to influence postoperative outcomes, secondary diagnoses and high-risk medications were also analyzed as contributing factors to CD°IV transfers. Regarding preexisting comorbidities, gastrointestinal disorders were identified as independent predictors for CD°IV transfers following TJAs, THAs, and TKAs. In addition, cardiological conditions proved to be independent predictors for CD°IV transfers after TJAs and TKAs, while pulmonary and neurological disorders were independent predictors for CD°IV transfers following THAs and TKAs.

Rheumatic medications, such as immunomodulatory drugs or corticosteroids, showed no correlation with CD°IV transfers, although other studies have demonstrated a correlation between underlying rheumatic diseases and postoperative complications [29, 30]. This is likely attributable to the small number of patients taking these medications (immunomodulatory drugs: *n* = 6; corticosteroid use: *n* = 6).

In our propensity score matching procedure, we intentionally did not include BMI as a matching variable, as we hypothesized that BMI might correlate with the clinical outcome and therefore wished to evaluate its influence separately. Contrary to our assumption, the BMI did not demonstrate any significant correlations with CD°IV transfers in our analysis.

To address the potential for residual confounding due to this omission—particularly given the possible interaction of BMI with frailty or comorbidities—a sensitivity analysis was performed. However, BMI did not show a significant association with the outcome in the subsequent multivariable regression model and did not change the estimates or significance of the other covariates. Both clinical experience and existing literature have well established that BMI is associated with severe complications following TJA [31, 32]. While BMI is frequently associated with an increased risk of postoperative complications, there is limited evidence directly linking BMI to ICU admissions. In our cohort, BMI was not a significant predictor of ICU transfer. This may be due to the relatively low number of patients with extreme obesity (e.g., BMI > 40) and the potential mitigating effects of standardized perioperative care protocols. Our findings suggest that BMI alone may not be a key determinant for ICU need in this clinical context.

The secondary outcome centered on identifying the most critical complications resulting in CD°IV ICU admissions. Cardiovascular events were most common, followed by neurovascular and pulmonary complications. Gastrointestinal and hemorrhagic events were less frequent, while rare cases involved urogenital conditions and isolated events such as severe intoxications, infections, or metabolic emergencies. These reasons for CD°IV transfers were extensively analyzed in this study (see Fig. 2) and are partially referenced in other research works [9, 18, 33]. The detailed presentation of ICU transfer causes can serve as an additional contribution to the understanding of these critical CD°IV events requiring intensive care.

This study has several notable strengths. The use of propensity score matching for age, sex, and Charlson Comorbidity Index (CCI) minimizes confounding factors, ensuring a robust comparison between groups. By focusing on severe, life-threatening complications (CD°IV) following total joint arthroplasty (TJA), the study addresses an underexplored yet clinically critical outcome. The integration of real-world data from a hospital database enhances the practical relevance of the findings, and the inclusion of comprehensive risk factors such as frailty, ASA scores, and preexisting conditions offers a holistic view of perioperative risks. Moreover, the identification of modifiable predictors, such as anticoagulant use, highlights actionable targets for improving patient care. The study’s consideration of both total hip arthroplasty (THA) and total knee arthroplasty (TKA) broadens its applicability across different types of joint replacements.

However, there are limitations that warrant consideration. The retrospective design inherently limits the ability to establish causation, and potential inaccuracies in data collection or missing parameters may introduce bias. Additionally, while the cohort size of CD°IV patients is reflective of real-world prevalence, it remains relatively small, potentially limiting the generalizability of the findings. The study was conducted at a single center, which may reduce external validity, as clinical protocols and patient populations vary across institutions. Residual confounding, despite matching, cannot be ruled out, particularly for unmeasured variables or socioeconomic aspects, lifestyle and other nonclinical factors. Finally, prospective, multicenter studies with larger sample sizes are needed to validate these results and further clarify the complex interplay of risk factors contributing to ICU transfers after TJA.

## Conclusions

This study provides important insights into determinants correlated with severe, life-threatening complications (CD°IV) requiring ICU admission after total joint arthroplasty (TJA). Using propensity score matching and comprehensive data analysis, we identified “frailty”, preexisting cardiological and gastrointestinal comorbidities, and the use of anticoagulants as independent predictors of ICU transfers following TJA. Medical complications—especially cardiological, pulmonary, renal, neurological, and gastrointestinal—seem to be drivers of adverse outcomes. The results highlight the complex interplay between patient vulnerability, preexisting conditions, and perioperative factors in determining ICU admissions. This study emphasizes the importance of prehabilitation, perioperative optimization, and multidisciplinary care in improving outcomes for high-risk patients. Variables such as BMI and rheumatic medications showed no significant associations, suggesting complex multifactorial interactions beyond this analysis.

Future prospective, multicenter studies are needed to validate these findings and develop targeted strategies to improve safety and outcomes in vulnerable TJA populations. This study contributes to the growing body of literature emphasizing the need for personalized approaches to orthopedic surgical care.

## Supplementary Information

Additional file 1

## Data Availability

The datasets used and/or analyzed during the current study are available from the corresponding author on reasonable request.

## Abbreviations

ICU: intensive care unit
OR: odds ratio
CI: confidence intervall
ICD-10: International Classification of Diseases, Tenth Revision
