# Supplementary material for: Intensive care needs after hip and knee replacement: understanding risk profiles for severe postoperative complications
Source: J Orthop Traumatol. 2025 Jul 3;26:42. doi: 10.1186/s10195-025-00862-x (PMC12229386; doi:10.1186/s10195-025-00862-x)
Supplement: Supplementary file 1 — Additional file 1 [file 10195_2025_862_MOESM1_ESM.docx]

**Intensive Care Needs After Hip and Knee Replacement: Understanding Risk Profiles for Severe Postoperative Complications**

**Abstract**

**Background:** The etiology of serious life-threatening events after total joint arthroplasty (TJA) is poorly elaborated and understood in the literature. The purpose of this study was to identify independent predictors of postoperative intensive care following total hip (THA) and total knee arthroplasty (TKA) and to clarify the circumstances leading to these transfers.

**Material and Methods:** One-hundred and forty-two patients suffering from postoperative intensive care-dependent serious adverse events (CD°IV) after THA or TKA were matched 1:1 with non-CD°IV patients using propensity score matching for age, sex, comorbidity (Charlson Comorbidity index, CCI) and year of treatment. Possible predictive factors for the need of postoperative intensive care were initially evaluated using univariate tests, followed by multivariate regression analyses to identify independent predictors.

**Results:** CD°IV transfers correlate with higher Hospitality Frailty Risk Score levels (HFRS) [mean 4.4 (SD 3.8) vs. mean 3.0 (SD 3.0); p < 0.001], higher ASA-Scores [mean 2.5 (SD 0.6) vs. mean 2.3 (SD 0.7); p = 0.02], a greater proportion of octogenarians [35.9% (n=51) vs. 23.9% (n=34); p = 0.028] and a higher incidence of medical complications [97.9% (n=139) vs. 60.6% (n=86); p < 0.001] compared to an adjusted control group after total joint arthroplasty (TJA).

Multivariate regression analysis confirmed “Frailty” (OR 1.14, 95% CI 1.05–1.23, p = .002), pre-existing cardiological (OR 2.0, 95% CI 1.004–4.1, p = 0.049) and gastrointestinal secondary diagnoses (OR 3.0, 95% CI 1.3–6.9, p = 0.01) and intake of anticoagulants (OR 2.7, 95% CI 1.6-4.6, p < 0.001) as independent risk factors for CD°IV ICU transfers after TJA.

**Conclusion:** Patients with CD°IV events after THA and TKA represent a complex, vulnerable and multimorbid patient population. There is a need for a multidisciplinary approach, that integrates pre-habilitation and perioperative risk assessments to reduce the occurrence of severe, life-threatening events requiring ICU care.

**Keywords**

Intensive Care, ICU, severe complications, Clavien-Dindo classification, TJA, TKA, THA, Arthroplasty, Frailty

**Level of Evidence**

Level III – retrospective cohort study

**Trial registration**

Retrospectively registered

**Introduction**

Osteoarthritis (OA) significantly impacts the lives of a substantial proportion of adults worldwide [1]. This growing burden is largely driven by the demographic shift and an aging population [2], which have led to an increasing demand for surgical interventions such as TJA and revision procedures[3, 4]. Despite advancements in surgical techniques and perioperative care [5, 6], a small but notable subset of patients experiences severe complications requiring admission to intensive care units (ICU) after TJA. The implementation of FAST-Track and specialized orthogeriatric (SOG) concepts in TJA represents an initial step towards safely guiding vulnerable patient populations through the surgical process [7, 8]. Nonetheless these unplanned admissions to the intensive care unit (ICU) following TJA occur in approximately 1 -12% [9–12], representing a resource and cost intensive aspect of postoperative care [13]. Despite the frequency of these severe events, the underlying risk profiles and mechanisms leading to ICU transfers remain inadequately understood, highlighting the need for further research.

The primary outcome of this study was to identify independent prognostic factors associated with life-threatening postoperative complications requiring ICU admission according to the Clavien-Dindo classification Grade IV (CD°IV) following total joint arthroplasty (TJA). Variables examined included the Hospital Frailty Risk Score (HFRS), the American Society of Anesthesiologists Physical Status Classification System (ASA), age, body mass index (BMI), type of arthroplasty (total hip or total knee arthroplasty), intra-hospital medical and surgical complications, comorbidities, and the use of potentially high-risk medications.

The Clavien-Dindo classification system grades complications on a scale of I to V based on the level of therapeutic intervention required, with CD°IV indicating life-threatening events that mandate intensive care management [14]. The secondary outcome was a detailed analysis of the most prevalent contributing factors among patients with CD°IV complications to better characterize the clinical profiles of high-risk patients and improve perioperative risk stratification.

**Materials and methods**

*Study Population and Data Collection*

This study is a retrospective matched-pair analysis derived from a database, generated from the hospital information system and the department’s joint registry. All patients who underwent primary elective total hip arthroplasty (THA) or total knee arthroplasty (TKA) between 2010 and 2023 were included, forming a consecutive series. Only primary joint arthroplasties were included in this study. Cases involving preoperative fractures, significant deformities, revision procedures or tumor endoprostheses were excluded from the analysis.

At last a total of 142 patients were identified in the in-house database using ICD-10 codes and Grade IV of the Clavien-Dindo classification (CD°IV) [14]. The Clavien-Dindo classification stratifies complications into five grades based on the required therapeutic intervention, with Grade IV representing severe, life-threatening conditions requiring intensive care management [14]. This indicates that the patients were transferred to an external intensive care unit to undergo specialized medical management, encompassing continuous monitoring and therapeutic interventions. As our department of anesthesiology keeps annual statistics on our patient transfers requiring intensive care, it was possible to check all 142 patients for completeness of data in the above-mentioned period. A full dataset was available for all patients included in the analysis. For all CD°IV cases, comprehensive review of both hospital discharge summaries and ICU-specific reports was conducted to confirm intensive care treatment and to determine the reasons for ICU admission. Diagnoses coded at the time of hospitalization and discharge were retrieved from the hospital information system (ORBIS®; Agfa Healthcare), including the corresponding ICD-10 codes. Coding was performed by professional clinical coders and subsequently verified by physicians using information from the patients’ medical records. Complications were assessed using ICD-10 codes documented at discharge. The Hospital Frailty Risk Score (HFRS) and Charlson Comorbidity Index (CCI) were calculated based on ICD-10 codes recorded at admission [15, 16]. Additional available data from the clinical information system included age, sex, body mass index (BMI), operative procedure, associated comorbidities, medications, length of hospital stay, time of surgery in minutes, CD°IV cases, medical and surgical complications, and reoperations within 90 days.

Propensity score matching (1:1) was conducted based on age, sex, and comorbidity (Charlson Comorbidity Index, CCI) and year of treatment. The final dataset comprised 284 patients: 142 requiring intensive care (CD°IV) and 142 matched non-CD°IV controls. Baseline characteristics were extracted from patient records.

*Endpoints*

The primary endpoint was the identification of CD°IV predictors. Additional data collected included associated comorbidities, revision surgeries within 90 days, and postoperative complications. Comorbidities and preoperative parameters recorded included cardiological, pulmonary, renal, gastrointestinal, neurological, oncological, and rheumatological diseases, as well as depression, diabetes, osteoporosis, preoperative anemia, malnutrition, nicotine abuse, chronically elevated CRP levels, and medications such as disease-modifying anti-rheumatic drugs (DMARDs), corticosteroids, platelet aggregation inhibitors (aspirin or P2Y12 antagonists), coumarin derivatives, or immunosuppressive agents. Perioperative complications were categorized into surgical complications (e.g., periprosthetic joint infection, periprosthetic fracture, wound healing disorders, hematoma, aseptic loosening and subsidence, dislocation, instability, wear of mobile components, arthrofibrosis, range-of-motion limitation, anterior knee pain, tendon rupture, and nerve injuries) and medical complications (e.g. cardiological, pulmonary, renal, thromboembolic, neurological, and cerebrovascular AEs, delirium, urinary tract infection, electrolyte imbalances, and anemia requiring transfusion). Complications were defined and assessed based on ICD-10 codes documented at discharge.

The secondary endpoint consisted of a detailed analysis of the primary causes leading to intensive care admissions among these patients.

*Surgical Techniques*

All procedures were performed within a single Department of Orthopedic Surgery at a University Medical Center. Patients received standardized treatment protocols for THA and TKA, including a unified rehabilitation program. The majority of patients underwent spinal anesthesia, with a minority receiving general or regional anesthesia. THA was performed in the lateral decubitus position using a minimally invasive anterolateral approach with cementless or cemented fixation. TKA procedures employed a cemented technique via a medial parapatellar approach without patellar resurfacing.

*Statistics*
Propensity score matching (PSM) was utilized to balance baseline characteristics between the CD°IV and non-CD°IV groups including age, sex, Charlson Comorbidity Index (CCI), and year of treatment. A nearest neighbor algorithm with a caliper width of 0.012 was used, chosen to ensure a high level of comparability between matched cases. Cases outside the region of common support were excluded.  PSM and statistical analysis was conducted using IBM SPSS Statistics (version 29.0.0) with a significance level set at p ≤ 0.05. Continuous variables are presented as means ± standard deviation (SD) and medians with interquartile ranges (IQR). Categorical data are reported as absolute (n) and percentage (%) frequencies. Continuous variables were tested for normal distribution using the Shapiro-Wilk test; as all variables were non-normally distributed, non-parametric methods such as the Mann-Whitney U test were applied for independent samples. Categorical variables were analyzed using the Chi-square test, and Fisher’s exact test was applied for small sample sizes or low expected frequencies. Multivariate logistic regression models were employed to identify independent predictors of postoperative CD°IV cases. Separate models were developed for preoperative and perioperative covariates, as well as for knee and hip arthroplasty, to account for the differing clinical and procedural factors influencing outcomes in these contexts. A forward selection procedure was applied, including variables with a univariate p-value < .10 in the model development process. These analyses facilitated the identification of confounders and independent predictors of CD°IV complications.

**Results**

*Baseline Characteristics:*

Between January 2010 and December 2023, a total of 34455 patients were identified through the local hospital computer database. Among these, 14505 cases underwent primary elective THA or TKA. Within this cohort 142 patients (0.98%) suffered from severe adverse events requiring ICU admission, classified as CD°IV. One patient had a deadly event and passed away after unsuccessful cardiopulmonary resuscitation.

The baseline characteristics of the CD°IV group and the control group, including age, sex, and Charlson Comorbidity Index (CCI), were well balanced through propensity score matching. Baseline characteristics are summarized in Table 1.

**Table 1: Baseline characteristics** **TJA:**

| **Total Joint Arthroplasty (TJA)** | | | | | |
| --- | --- | --- | --- | --- | --- |
|  | **Controls**  (n=142) | **CD°IV Patients**  (n=142) | | **total population**  (n=284) | **p-value** |
| **♀** n (%) | 98 (69.0) | 94 (66.2) | | 192 (67.6) | .612* |
| **♂** n (%) | 44 (31.0) | 48 (33.8) | | 92 (32.4) | .612* |
| **Age (years)**  Mean (SD)  Median (IQR) | 73.4 (8.6)  74.0 (10.0) | 75.1 (8.8)  77.0 (13.0) | | 74.2 (8.8)  76.0 (11) | .064** |
|  |  |  | |  |  |
| **CCI**  Mean (SD)  Median (IQR) | 3.6 (1.7)  3.0 (1.0) | 3.8 (1.5)  4.0 (1.0) | | 3.7 (1.6)  3.5 (1.0) | .179** |
| **THA** n (%) | 66 (46.5) | 78 (54.9) | | 144 (50.7) | .154* |
| **TKA** n (%) | 76 (53.5) | 64 (45.1) | | 140 (49.3) | .154* |
|  |  |  | |  |  |
| **Side left** n (%) | 56 (39.4) | | 67 (47.2) | 123 (43.3) | .120** |
| **Side right** n (%)  **Side both** n (%) | 86 (60.6)  0 (0) | | 73 (51.4)  2 (1.4) | 159 (56.0)  2 (0.7) |  |
| **BMI**  Mean (SD)  Median (IQR)  **Adipositas (yes/no)**  n (%)  **Adipositas ≥ II**  n (%) | 29.6 (6.7)  28.8 (9.3)  64 (63.4)  24 (39.3) | 29.1 (6.1)  27.9 (7.8)  58 (60.4)  27 (41.5) | | 29.3 (6.4)  28.5 (8.7)  122 (61.9)  51 (40.5) | .541**  .670*  .802* |
| **HFRS**  Mean (SD)  Median (IQR) | 3.0 (3.0)  2.3 (2.3) | 4.4 (3.8)  3.2 (4.8) | | 3.7 (3.5)  2.3 (3.4) | **<.001**** |
| **ASA – Score**  Mean (SD)  Median (IQR) | 2.3 (0.7)  2.0 (1.0) | 2.5 (0.6)  3.0 (1.0) | | 2.4 (0.6)  2.0 (1.0) | **.020**** |
| **LOS (days)**  Mean (SD)  Median (IQR) | 9.4 (3.2)  9.0 (3.0) | 4.9 (4.5)  4.0 (4.0) | | 7.2 (4.5)  7.0 (5.0) | **<.001**** |
| **time of surgery (minutes)**  Mean (SD)  Median (IQR) | 75.5 (21.6)  74.0 (29.0) | 78.1 (25.0)  73.0 (30.3) | | 76.8 (23.4)  73.5 (29.5) | .594** |

Clavien-Dindo IV (CD°IV) = life threatening complications leading to transfer to an intermediate care unit or intensive care unit, CCI= Charlson Comorbidity Index, TJA = Total Joint Arthroplasty, THA = Total hip arthroplasty, TKA = Total knee arthroplasty, Age in years, BMI = body mass index, Adipositas (yes/no) = BMI ≥ 30 vs. normal weight patients, Adipositas ≥ II = BMI ≥ 35 vs. normal weight patients, ASA = American Society of Anesthesiologists, LOS = length of stay in days, time of surgery in minutes, bold values = p ≤ 0.05; *Chi-square-test. **Mann-Whitney-U-test.

*Primary Outcome: Influencing Factors on CD°IV Transfers*

*“Frailty”:*

The CD°IV cohort across all TJA cases demonstrated a significantly higher proportion of "Frailty" based on the HFRS compared to the control group, with a mean score of 4.4 (SD 3.8) versus 3.0 (SD 3.0) (p < 0.001). These findings were also confirmed for both THA [mean 4.9 (SD 4.4) vs. mean 3.3 (SD 3.5); p = 0.012] and TKA [mean 3.7 (SD 2.6) vs. mean 2.8 (SD 2.5); p = 0.008]. Figure 1 shows a graphical representation.

**Figure 1: HFRS values in CD°IV patients vs. control group after TJA, THA and TKA
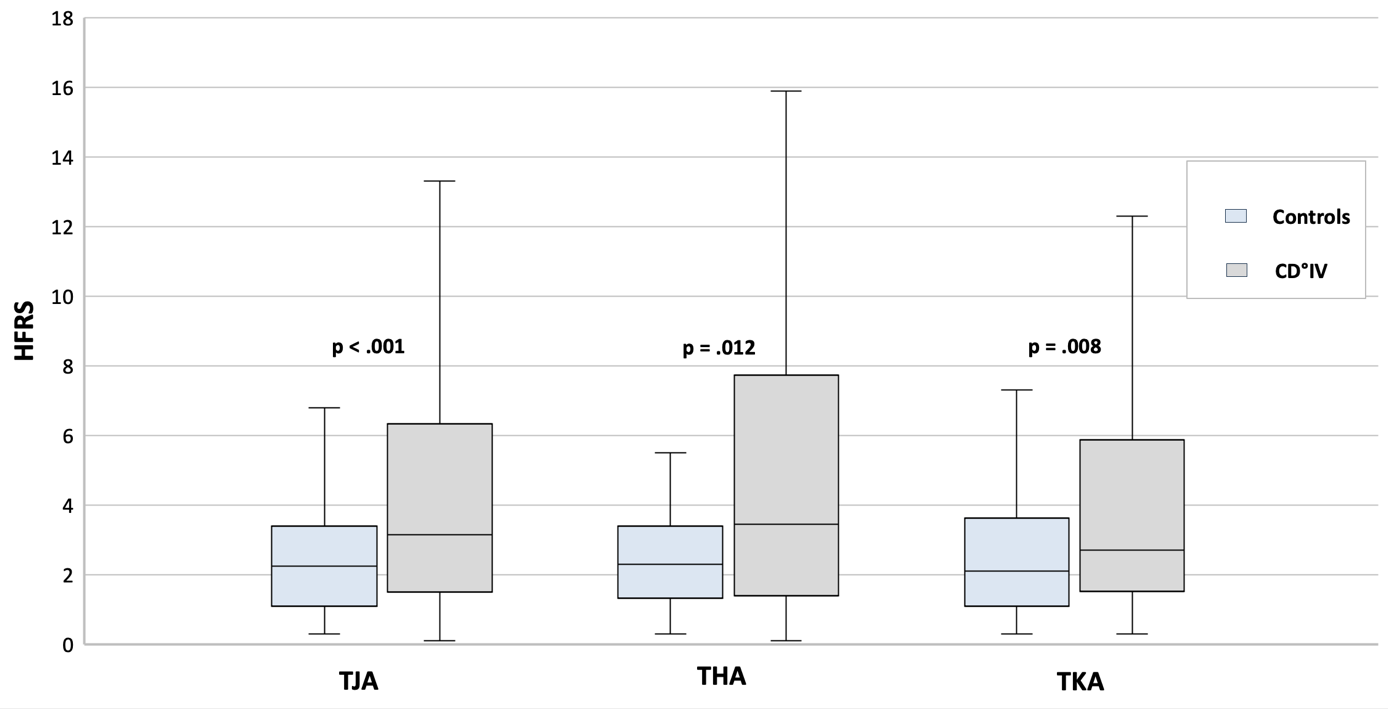
**

HFRS = Hospitality Frailty Risk Score, THA = Total Hip Arthroplasty, TKA = Total Knee Arthroplasty, TJA = Total Joint Arthroplasty, CD°IV = Severe life-threatening complications requiring ICU transfer according to Clavien-Dindo Classification Grade IV

In the multivariate analysis, the HFRS was confirmed as an independent influencing factor for a transfer to CD°IV after all TJAs (OR 1.14, 95% CI 1.05–1.23, p = .002) and after THA (OR 1.12, 95% CI 1.01–1.23, p = .024).

*Octogenarians (Age ≥ 80 years):*

A closer look at the group of over 80 year old patients revealed a higher rate of CD°IV transfers than in the control group after TJA [35.9% (n=51) vs. 23.9% (n=34); p = 0.028]. This also applies to the TKA group [40.6% (n=26) vs. 23.7% (n=18); p = 0.031].

*ASA-Score:*

The Analysis of the ASA score indicated a poorer preoperative physical health status among CD°IV patients compared to the control group in all TJA patients [mean 2.5 (SD 0.6) vs. mean 2.3 (SD 0.7); p = 0.02] and THA patients [mean 2.6 (SD 0.5) vs. mean 2.3 (SD 0.7); p = 0.004].

*Complications:*

- *Surgical Complications:*

There was no association between CD°IV transfer and pooled surgical complications. In detail postoperative hematoma was the only surgical factor associated with an increased incidence of CD°IV transfers for all TJA [12.0% (n=17) vs. 3.5% (n=5); p = 0.008], as well as for THA [12.8% (n=10) vs. 3.0% (n=2); p = 0.034].

- *Medical Complications:*

The pooled medical complications demonstrated significantly higher rates of ICU transfers according to CD°IV compared to the control group in the univariate analysis after TJA [97.9% (n=139) vs. 60.6% (n=86); p < 0.001], but also after THA [100% (n=78) vs. 66.7% (n=44); p < 0.001] and TKA [95.3% (n=61) vs. 55.3% (n=42); p < 0.001]. The leading causes of these medical complications after TJA are comprehensively detailed in Figure 2 and Appendix 1 for further evaluation.

**Figure 2: Medical Complications after TJA**


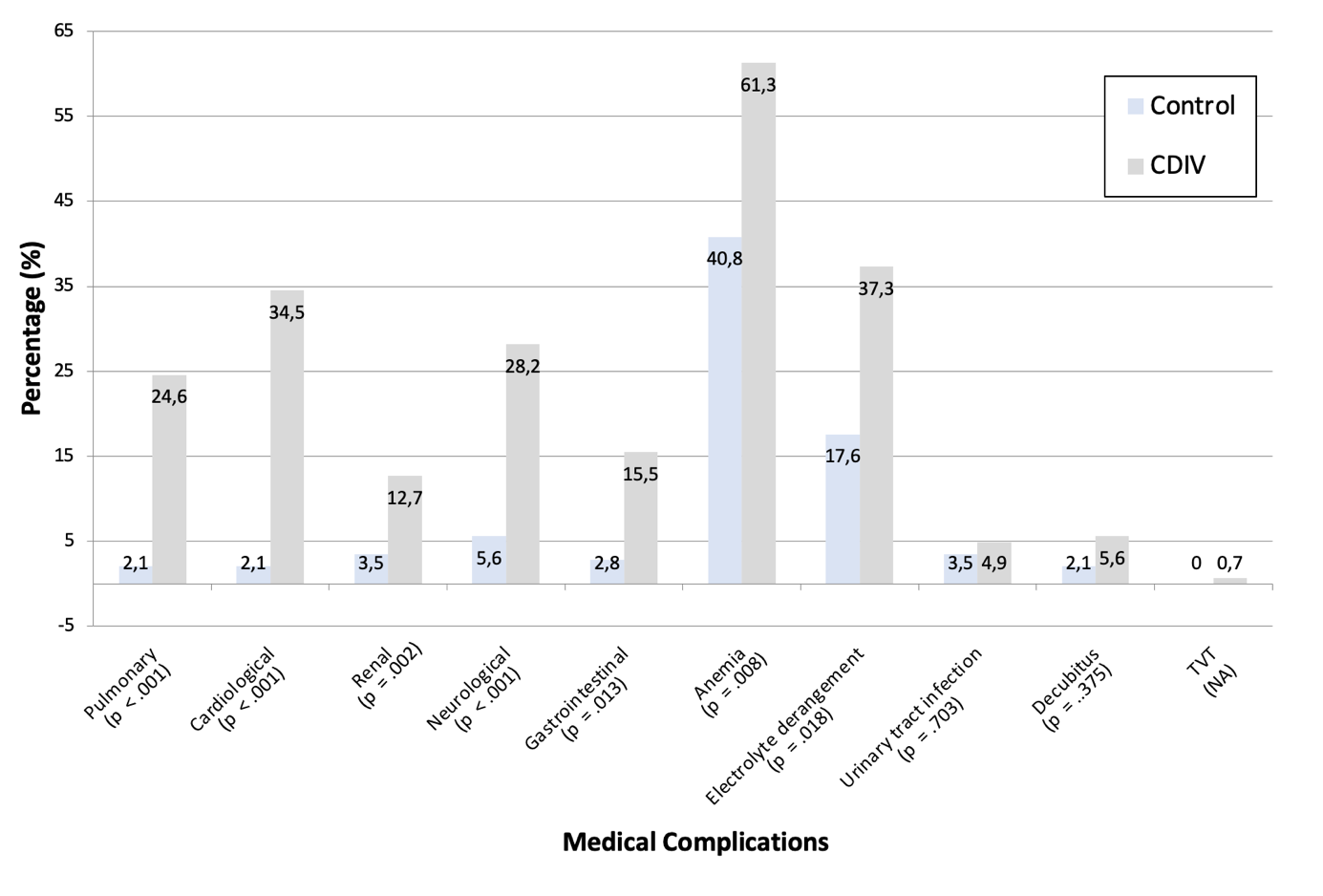


TJA = Total joint arthroplasty, Clavien-Dindo IV (CD°IV) = life threatening complications leading to transfer to an intermediate care unit or intensive care unit

In the multivariate analyses, independent medical predictors for CD°IV ICU transfers following TJA were identified. Independent predictors were pulmonary complications (OR 42.2, 95% CI 11.0–161.6, p < 0.001), cardiological complications (OR 101.9, 95% CI 27.4–379.2, p < 0.001), renal complications (OR 15.6, 95% CI 4.7–51.9, p < 0.001), neurological complications (OR 20.1, 95% CI 7.5–53.6, p < 0.001), gastrointestinal complications (OR 31.4, 95% CI 9.1–107.8, p < 0.001) and postoperative anemia (OR 2.1, 95% CI 1.0–4.3, p = 0.047).

A detailed presentation of the multivariate analysis can be found in Appendix 2.

*Comorbidities and potentially risk generating medication:*

*Secondary diagnoses:*

In univariate analysis, patients with CD°IV transfers exhibited significantly higher rates of cardiological (88.0 % vs. 76.1 %, p = 0.009), neurovascular (29.6 % vs. 17.6 %, p = 0.018), haemato‐oncological (23.2 % vs. 14.1 %, p = 0.048), and gastrointestinal comorbidities (17.6 % vs. 6.3 %, p = 0.003) compared to controls.

*Potentially risk generating medication:*

Anticoagulant use was also significantly elevated in CD°IV patients (48.6 % vs. 23.9 %, p < 0.001).

Appendix 3 and 4 provide an overview of the secondary diagnoses and the potentially risk generating medication, that correlate significantly with an ICU transfer after CD°IV.

The multivariate calculations of the above-mentioned influencing variables revealed the following independent predictors for CD°IV ICU transfers.

*Secondary diagnoses:*

Independent predictors of CD°IV after TJA were cardiological concomitant diseases (OR 2.0, 95% CI 1.004–4.1, p = 0.049) and gastrointestinal comorbidities (OR 3.0, 95% CI 1.3–6.9, p = 0.01). A detailed presentation of the multivariate analysis can be found in Appendix 5.

*Potentially risk generating medication:*

Pooled anticoagulant intake was an independent predictor for CD°IV transfers after TJA (OR 2.7, 95% CI 1.6-4.6, p < 0.001), THA (OR 2.3, 95% CI 1.1-4.8, p = 0.023) and TKA (OR 4.1, 95% CI 1.9-8.6, p < 0.001).

Figure 3 shows a visual summary of the most important significant results of all multivariate logistic regression models according to TJA graphically presented in a forest plot.

**Figure 3: Independent Predictors for CD°IV transfers after TJA**


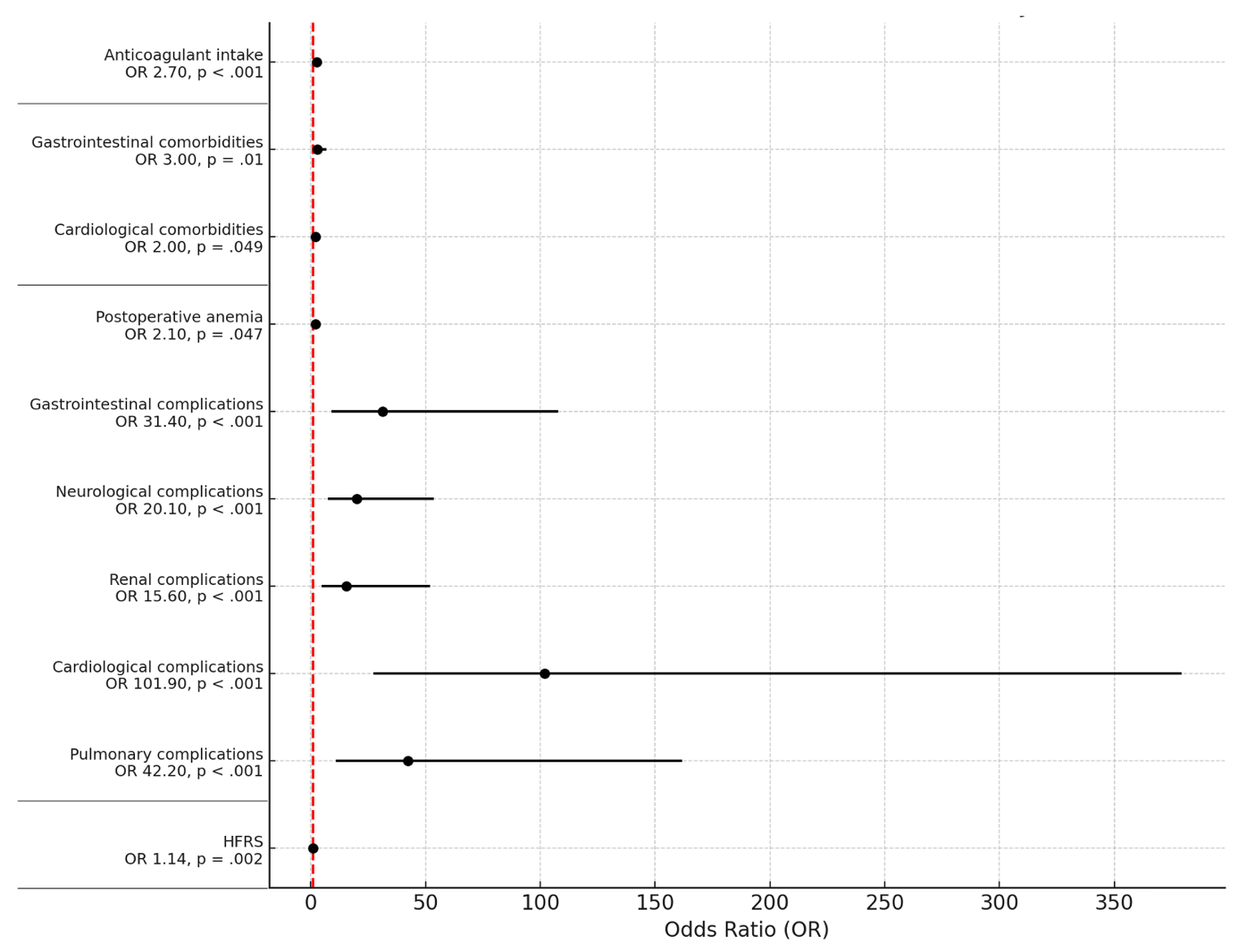


TJA = Total joint arthroplasty, Clavien-Dindo IV (CD°IV) = life threatening complications leading to transfer to an intermediate care unit or intensive care unit, HFRS = Hospitality Frailty Risk Score, OR = Odds Ratio, red dashed line = marks a OR of 1

*Secondary Outcome: Analysis of the underlying causes for CD°IV transfers necessitating intensive care*

The secondary outcome focused on identifying the key drivers of CD°IV transfers requiring intensive care. Cardiological events represent the primary cause of CD°IV transfers, comprising approximately one-third of all cases. The most common cardiological reasons, in descending order, include acute myocardial infarction, acute coronary syndromes, arrhythmias (e.g., tachyarrhythmia absoluta, newly developed atrial fibrillation, etc.), cardiac decompensation and hypertensive crisis.

Neurovascular events and pulmonary complications follow as the second and third most frequent reasons, respectively. Neurological serious adverse events (SAEs) include transient ischemic attacks (TIA)/strokes, exacerbated severe delirium, severe epileptic seizures, and other neurological causes. Pulmonary SAEs mainly consist of severe pneumonia, pulmonary embolism, and severe respiratory decompensation.

The fourth most common cause of CD° IV transfers are gastrointestinal events. These include symptoms of an acute abdomen, such as (sub-)ileus events, cholecystolithiasis requiring endoscopic retrograde cholangiopancreatography (ERCP), incarceration due to recurrent incisional hernia, upper gastrointestinal bleeding, severe gastritis, and one case of pancolitis related to a severe *Clostridium difficile* infection.

Approximately 10% of cases are attributed to hemorrhagic events, consisting of severe hemorrhages requiring vascular surgical intervention and hemorrhagic shock resulting from acute bleeding.

Urogenital events and "other" causes account for a minor proportion of cases. "Other" events include single cases such as severe opioid intoxication with respiratory insufficiency, severe drug intoxication resulting in transient tetraparesis, agranulocytosis following metamizole use, severe drug-induced hyponatremia, severe blood pressure dysregulation associated with pheochromocytoma, severe sepsis with multiorgan failure (renal insufficiency requiring dialysis, myocardial infarction, and severe pneumonia), and severe COVID-19 infection during the pandemic. Figure 4 shows the most common reasons for CD°IV relocations.

**Figure 4: Main Causes for CD°IV transfers to an ICU**


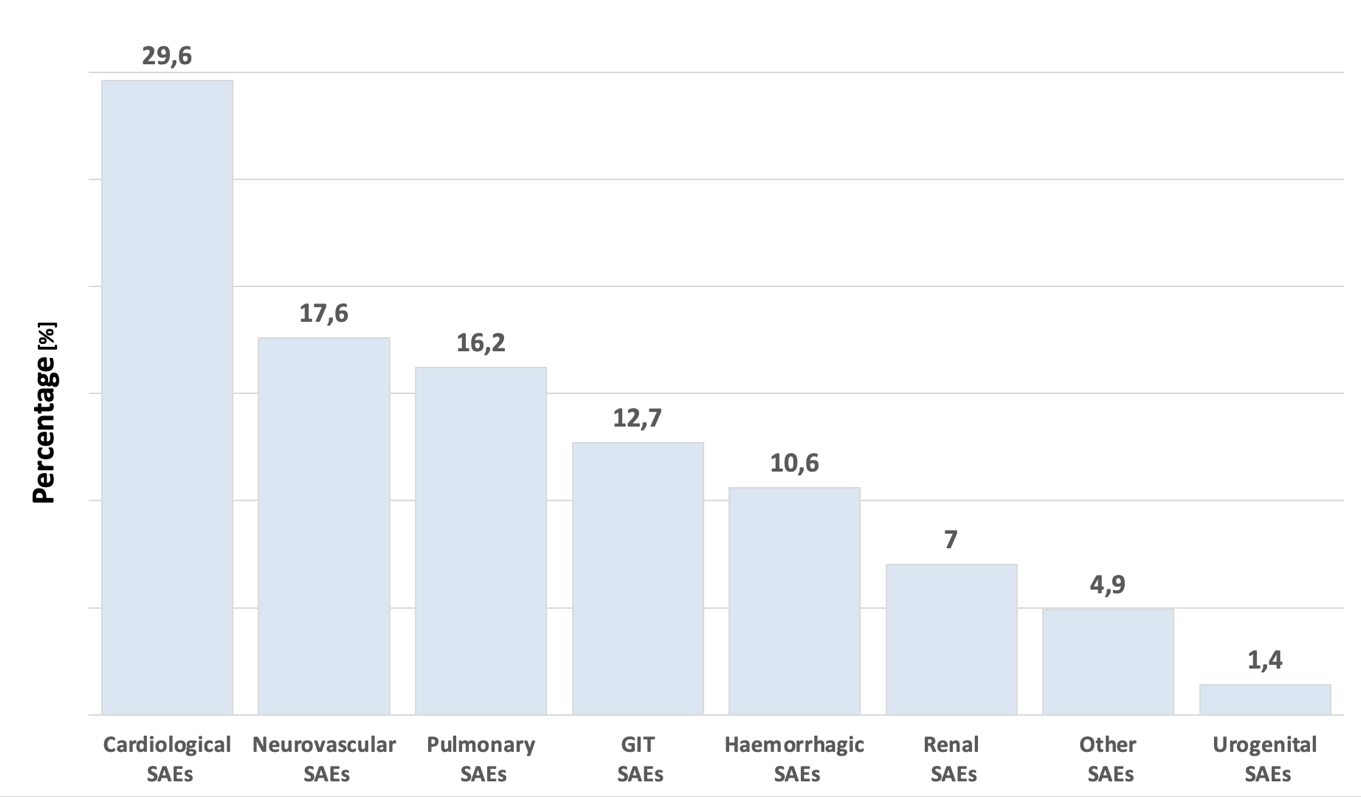


n = 142 CD°IV transfers, SAE = Serious adverse event, GIT = Gastro-intestinal;

**Discussion**

After the matching process, the groups exhibited a well-balanced and comparable distribution in terms of age, sex, BMI, type of arthroplasty (THA or TKA), and time of surgery in minutes. The patient population was characterized by overweight tendencies, with an average BMI of 29.3 (classified as pre-obesity), a relatively advanced mean age of 74 years, and a substantial burden of comorbidities, reflected in an average CCI of 3.7. Due to the ICU transfers, CD°IV patients exhibited a shorter average hospital stay, at 4.9 days. The overall mean LOS across the cohort was 7.2 days, showing a more accurately reflection of routine clinical practice in Germany [17]. The overall cohort consisted predominantly of female patients, with a ratio of approximately 2:1 in CD°IV patients compared to the control group. Courtney et al. also reported higher rates of ICU admissions among women in their research. However, the literature does not support this finding as an independent predictor [18]. With a rate of approximately 1% CD°IV transfers in our patient cohort, this percentage falls at the lower end of the range of 1 - 12% [9–12] reported in the literature. This also offers a realistic representation of routine clinical practice.

Meyer et al. (2020) demonstrated in retrospective analysis that a high HFRS constitutes an independent risk factor for adverse outcomes following TJA, including reoperations (OR 2.1, 95% CI 1.46-3.09, p < .001) and complications (internal complications: OR 3.72; 95% CI, 2.28-6.08, p < .001; surgical complications: OR = 3.74, 95% CI, 2.41-5.82, p < .001) [19]. Extending these findings, our analysis identified the HFRS as an independent predictor of CD°IV ICU transfers following TJA (OR 1.14; 95% CI, 1.05–1.23; p = .002). While the endpoints differ, our results contribute novel insights to the current body of evidence and underscore the clinical relevance of frailty assessment in this context.

In the field of orthogeriatrics significant advancements have been made in research over the past years [20, 21]. Specialized orthogeriatric concepts focus on pre-habilitation, geriatric-internal medicine, and intensive physiotherapeutic care in combination with FAST- Track concepts to improve postoperative outcomes following TJA. Multiple studies have shown that "frailty" is an independent predictor for postoperative complications after primary TJA [22–25]. The results of the present study confirmed this finding for severe intensive-care complications classified as CD°IV. Additionally, the age group of octogenarians was identified as a possible predictor for these transfers. These findings add valuable insights to the existing literature, particularly by highlighting the role of “frailty” and advanced age as important predictors in orthogeriatric outcomes.

Focusing on the ASA score, previous studies have demonstrated that patients preoperatively classified as ASA 3 and 4 experience significantly higher rates of intraoperative and postoperative complications, as well as prolonged intensive care unit (ICU) stays. Our findings corroborate these results [26].

Postoperative complications showed distinct patterns between surgical and medical events. Our findings revealed no increased incidence of surgical complications within the CD°IV transfer group. We hypothesize that while surgical complications may contribute to adverse outcomes, their severity in our cohort did not necessitate ICU transfers. Specifically, only postoperative hematomas were significantly correlated with CD°IV transfers. This may also be associated with the use of anticoagulants and postoperative anemia, as both emerged as independent predictors of CD°IV transfers for all TJAs, consistent with the current literature [9].

A closer examination of the medical complications indicates a significant association with CD°IV transfers. In particular, pulmonary, cardiological, renal, neurological, and gastrointestinal complications were identified as independent predictors of CD°IV transfers. The wide confidence intervals and extremely high odds ratio for cardiological complications reflect a limited number of events and high variability in this subgroup. Although the association remained statistically significant, the magnitude of the effect should be interpreted with caution. These results supports the outcome of other studies and expands the scope of results [27–29]. Although both medical and surgical complications appear to be relevant components contributing to ICU transfers, further clinical research is necessary to differentiate their specific impact and to develop targeted strategies for early identification and risk mitigation. In clinical reality, ICU transfers are often triggered by a cumulative burden of complications rather than a single isolated event. Future research should aim to model these interactions more precisely. Given their potential to influence postoperative outcomes, secondary diagnoses and high-risk medications were also analyzed as contributing factors to CD°IV transfers. Regarding pre-existing comorbidities, gastrointestinal disorders were identified as independent predictors for CD° IV transfers following TJAs, THAs, and TKAs. In addition, cardiological conditions proved to be independent predictors for CD° IV transfers after TJAs and TKAs, while pulmonary and neurological disorders were independent predictors for CD° IV transfers following THAs and TKAs.

Rheumatic medications, such as immunomodulatory drugs or corticosteroids, showed no correlation with CD° IV transfers, although other studies have demonstrated a correlation between underlying rheumatic diseases and postoperative complications [30, 31]. This is likely attributable to the small number of patients taking these medications (immunomodulatory drugs: n=6; corticosteroid use: n=6).

In our propensity score matching procedure, we intentionally did not include BMI as a matching variable, as we hypothesized that BMI might correlate with the clinical outcome and therefore wished to evaluate its influence separately. Contrary to our assumption the BMI did not demonstrate any significant correlations with CD° IV transfers in our analysis.

To address the potential for residual confounding due to this omission—particularly given the possible interaction of BMI with frailty or comorbidities—a sensitivity analysis was performed. However, BMI did not show a significant association with the outcome in the subsequent multivariable regression model and did not change the estimates or significance of the other covariates. Both clinical experience and existing literature have well established that BMI is associated with severe complications following TJA [32, 33]. While BMI is frequently associated with an increased risk of postoperative complications, there is limited evidence directly linking BMI to ICU admissions. In our cohort, BMI was not a significant predictor of ICU transfer. This may be due to the relatively low number of patients with extreme obesity (e.g., BMI > 40), and the potential mitigating effects of standardized perioperative care protocols. Our findings suggest that BMI alone may not be a key determinant for ICU need in this clinical context.

The secondary outcome centered on identifying the most critical complications resulting in CD°IV ICU admissions. Cardiovascular events were most common, followed by neurovascular and pulmonary complications. Gastrointestinal and hemorrhagic events were less frequent, while rare cases involved urogenital conditions and isolated events such as severe intoxications, infections, or metabolic emergencies. These reasons for CD°IV transfers were extensively analyzed in this study (see figure 2) and are partially referenced in other research works [9, 18, 34]. The detailed presentation of ICU transfer causes can serve as an additional contribution to the understanding of these critical CD° IV events requiring intensive care.

This study has several notable strengths. The use of propensity score matching for age, sex, and Charlson Comorbidity Index (CCI) minimizes confounding factors, ensuring a robust comparison between groups. By focusing on severe, life-threatening complications (CD°IV) following total joint arthroplasty (TJA), the study addresses an underexplored yet clinically critical outcome. The integration of real-world data from a hospital database enhances the practical relevance of the findings, and the inclusion of comprehensive risk factors such as frailty, ASA scores, and pre-existing conditions offers a holistic view of perioperative risks. Moreover, the identification of modifiable predictors, such as anticoagulant use, highlights actionable targets for improving patient care. The study’s consideration of both total hip arthroplasty (THA) and total knee arthroplasty (TKA) broadens its applicability across different types of joint replacements.

However, there are limitations that warrant consideration. The retrospective design inherently limits the ability to establish causation, and potential inaccuracies in data collection or missing parameters may introduce bias. Additionally, while the cohort size of CD°IV patients is reflective of real-world prevalence, it remains relatively small, potentially limiting the generalizability of the findings. The study was conducted at a single center, which may reduce external validity, as clinical protocols and patient populations vary across institutions. Residual confounding, despite matching, cannot be ruled out, particularly for unmeasured variables or socioeconomic aspects, lifestyle and other non-clinical factors. Finally, prospective, multicenter studies with larger sample sizes are needed to validate these results and further clarify the complex interplay of risk factors contributing to ICU transfers after TJA.

**Conclusion**

This study provides important insights into determinants correlated with severe, life-threatening complications (CD°IV) requiring ICU admission after total joint arthroplasty (TJA). Using propensity score matching and comprehensive data analysis, we identified “frailty”, pre-existing cardiological and gastrointestinal comorbidities, and the use of anticoagulants as independent predictors of ICU transfers following TJA. Medical complications - especially cardiological, pulmonary, renal, neurological, and gastrointestinal - seem to be drivers of adverse outcomes. The results highlight the complex interplay between patient vulnerability, pre-existing conditions, and perioperative factors in determining ICU admissions. This study emphasizes the importance of pre-habilitation, perioperative optimization, and multidisciplinary care in improving outcomes for high-risk patients. Variables like BMI and rheumatic medications showed no significant associations, suggesting complex multifactorial interactions beyond this analysis.

Future prospective, multicenter studies are needed to validate these findings and develop targeted strategies to improve safety and outcomes in vulnerable TJA populations. This study contributes to the growing body of literature emphasizing the need for personalized approaches to orthopedic surgical care.

**References**

1. Vina, E. R., & Kwoh, C. K. (2018). Epidemiology of osteoarthritis: literature update. *Current Opinion in Rheumatology*, *30*(2), 160–167. https://doi.org/10.1097/BOR.0000000000000479

2. Steinmetz, J. D., Culbreth, G. T., Haile, L. M., Rafferty, Q., Lo, J., Fukutaki, K. G., … Kopec, J. A. (2023). Global, regional, and national burden of osteoarthritis, 1990–2020 and projections to 2050: a systematic analysis for the Global Burden of Disease Study 2021. *The Lancet Rheumatology*, *5*(9), e508–e522. https://doi.org/10.1016/S2665-9913(23)00163-7

3. Sloan, M., Premkumar, A., & Sheth, N. P. (2018). Projected Volume of Primary Total Joint Arthroplasty in the U.S., 2014 to 2030. *Journal of Bone and Joint Surgery*, *100*(17), 1455–1460. https://doi.org/10.2106/JBJS.17.01617

4. Rupp, M., Lau, E., Kurtz, S. M., & Alt, V. (2020). Projections of Primary TKA and THA in Germany From 2016 Through 2040. *Clinical Orthopaedics & Related Research*, *478*(7), 1622–1633. https://doi.org/10.1097/CORR.0000000000001214

5. MacMahon, A., Rao, S. S., Chaudhry, Y. P., Hasan, S. A., Epstein, J. A., Hegde, V., … Khanuja, H. S. (2022). Preoperative Patient Optimization in Total Joint Arthroplasty—The Paradigm Shift from Preoperative Clearance: A Narrative Review. *HSS Journal®: The Musculoskeletal Journal of Hospital for Special Surgery*, *18*(3), 418–427. https://doi.org/10.1177/15563316211030923

6. Johns, W. L., Layon, D., Golladay, G. J., Kates, S. L., Scott, M., & Patel, N. K. (2020). Preoperative Risk Factor Screening Protocols in Total Joint Arthroplasty: A Systematic Review. *The Journal of Arthroplasty*, *35*(11), 3353–3363. https://doi.org/10.1016/j.arth.2020.05.074

7. Greimel, F., Schiegl, J., Meyer, M., Grifka, J., & Maderbacher, G. (2024). Fast-Track-Endoprothetik. *Die Orthopädie*, *53*(2), 117–126. https://doi.org/10.1007/s00132-023-04465-4

8. Kappenschneider, T., Bammert, P., Maderbacher, G., Greimel, F., Holzapfel, D. E., Schwarz, T., … Meyer, M. (2023). The impact of elective total hip and knee arthroplasty on physical performance in orthogeriatric patients: a prospective intervention study. *BMC geriatrics*, *23*(1), 763. https://doi.org/10.1186/s12877-023-04460-6

9. Sukhonthamarn, K., Grosso, M. J., Sherman, M. B., Restrepo, C., & Parvizi, J. (2020). Risk Factors for Unplanned Admission to the Intensive Care Unit After Elective Total Joint Arthroplasty. *The Journal of Arthroplasty*, *35*(7), 1937–1940. https://doi.org/10.1016/j.arth.2020.03.003

10. Parvizi, J., Pour, A. E., Peak, E. L., Sharkey, P. F., Hozack, W. J., & Rothman, R. H. (2006). One-Stage Bilateral Total Hip Arthroplasty Compared With Unilateral Total Hip Arthroplasty. *The Journal of Arthroplasty*, *21*(6), 26–31. https://doi.org/10.1016/j.arth.2006.04.013

11. Memtsoudis, S. G., Sun, X., Chiu, Y.-L., Nurok, M., Stundner, O., Pastores, S. M., & Mazumdar, M. (2012). Utilization of Critical Care Services among Patients Undergoing Total Hip and Knee Arthroplasty: Epidemiology and Risk Factors. *Anesthesiology*, *117*(1), 107–116. https://doi.org/10.1097/ALN.0b013e31825afd36

12. Price, V., Thuraisingam, S., Choong, P. F., Perriman, D., & Dowsey, M. M. (2024). Does admission to intensive care post total joint arthroplasty result in poorer outcomes 12‐months after surgery? *ANZ Journal of Surgery*, ans.19294. https://doi.org/10.1111/ans.19294

13. Baumgartner, B. T., Karas, V., Kildow, B. J., Cunningham, D. J., Klement, M. R., Green, C. L., … Seyler, T. M. (2018). Inpatient Consults and Complications During Primary Total Joint Arthroplasty in a Bundled Care Model. *The Journal of Arthroplasty*, *33*(4), 973–975. https://doi.org/10.1016/j.arth.2017.11.042

14. Dindo, D., Demartines, N., & Clavien, P.-A. (2004). Classification of Surgical Complications: A New Proposal With Evaluation in a Cohort of 6336 Patients and Results of a Survey. *Annals of Surgery*, *240*(2), 205–213. https://doi.org/10.1097/01.sla.0000133083.54934.ae

15. Gilbert, T., Neuburger, J., Kraindler, J., Keeble, E., Smith, P., Ariti, C., … Conroy, S. (2018). Development and validation of a Hospital Frailty Risk Score focusing on older people in acute care settings using electronic hospital records: an observational study. *The Lancet*, *391*(10132), 1775–1782. https://doi.org/10.1016/S0140-6736(18)30668-8

16. Charlson, M. E., Carrozzino, D., Guidi, J., & Patierno, C. (2022). Charlson Comorbidity Index: A Critical Review of Clinimetric Properties. *Psychotherapy and Psychosomatics*, *91*(1), 8–35. https://doi.org/10.1159/000521288

17. Kostev, K., Gyasi, R. M., Konrad, M., Yon, D. K., & Jacob, L. (2024). Hospital Length of Stay and Associated Factors in Patients with Osteoarthritis from Germany: A Cross-Sectional Study. *Journal of Clinical Medicine*, *13*(9), 2628. https://doi.org/10.3390/jcm13092628

18. Courtney, P. M., Melnic, C. M., Gutsche, J., Hume, E. L., & Lee, G.-C. (2015). Which patients need critical care intervention after total joint arthroplasty?: a prospective study of factors associated with the need for intensive care following surgery. *The Bone & Joint Journal*, *97-B*(11), 1512–1518. https://doi.org/10.1302/0301-620X.97B11.35813

19. Meyer, M., Parik, L., Leiß, F., Renkawitz, T., Grifka, J., & Weber, M. (2020). Hospital Frailty Risk Score Predicts Adverse Events in Primary Total Hip and Knee Arthroplasty. *The Journal of Arthroplasty*, *35*(12), 3498-3504.e3. https://doi.org/10.1016/j.arth.2020.06.087

20. Flexman, A. M., Ryerson, C. J., Augustin, T., & Walsh, R. M. (o. J.). Frailty and postoperative outcomes in patients undergoing surgery for degenerative spine disease, 1.

21. Schmucker, A. M., Hupert, N., & Mandl, L. A. (2019). The Impact of Frailty on Short-Term Outcomes After Elective Hip and Knee Arthroplasty in Older Adults: A Systematic Review. *Geriatric Orthopaedic Surgery & Rehabilitation*, *10*, 215145931983510. https://doi.org/10.1177/2151459319835109

22. Meyer, M., Parik, L., Leiß, F., Renkawitz, T., Grifka, J., & Weber, M. (2020). Hospital Frailty Risk Score Predicts Adverse Events in Primary Total Hip and Knee Arthroplasty. *The Journal of Arthroplasty*, *35*(12), 3498-3504.e3. https://doi.org/10.1016/j.arth.2020.06.087

23. Wen, H., Liu, T., & Li, J. (2023). Association between frailty and clinical post-operative outcomes in patients following hip arthroplasty: a systematic review and meta-analysis. *International Orthopaedics*, *47*(3), 667–675. https://doi.org/10.1007/s00264-022-05657-x

24. Kappenschneider, T., Bammert, P., Maderbacher, G., Greimel, F., Parik, L., Holzapfel, D. E., … Meyer, M. (2024). The impact of primary total hip and knee replacement on frailty: an observational prospective analysis. *BMC Musculoskeletal Disorders*, *25*(1), 78. https://doi.org/10.1186/s12891-024-07210-w

25. Tram, M. K., Tabbaa, A., Lakra, A., Anoushiravani, A. A., Bernasek, T. L., Lyons, S. T., & O’Connor, C. M. (2024). Patient Frailty is Correlated With Increased Adverse Events and Costs After Revision Total Hip Arthroplasty. *The Journal of Arthroplasty*, *39*(5), 1151-1156.e4. https://doi.org/10.1016/j.arth.2023.12.027

26. Pflüger, M. J., Frömel, D. E., & Meurer, A. (2021). Total Hip Arthroplasty Revision Surgery: Impact of Morbidity on Perioperative Outcomes. *The Journal of Arthroplasty*, *36*(2), 676–681. https://doi.org/10.1016/j.arth.2020.08.005

27. Courtney, P. M., Whitaker, C. M., Gutsche, J. T., Hume, E. L., & Lee, G.-C. (2014). Predictors of the need for critical care after total joint arthroplasty: an update of our institutional risk stratification model. *The Journal of Arthroplasty*, *29*(7), 1350–1354. https://doi.org/10.1016/j.arth.2014.02.028

28. Durairaj, L., Will, J. G., Torner, J. C., & Doebbeling, B. N. (2003). Prognostic factors for mortality following interhospital transfers to the medical intensive care unit of a tertiary referral center. *Critical Care Medicine*, *31*(7), 1981–1986. https://doi.org/10.1097/01.ccm.0000069730.02769.16

29. Kay, H. F., Chotai, S., Wick, J. B., Stonko, D. P., McGirt, M. J., & Devin, C. J. (2016). Preoperative and surgical factors associated with postoperative intensive care unit admission following operative treatment for degenerative lumbar spine disease. *European Spine Journal*, *25*(3), 843–849. https://doi.org/10.1007/s00586-015-4175-8

30. Ravi, B., Escott, B., Shah, P. S., Jenkinson, R., Chahal, J., Bogoch, E., … Hawker, G. (2012). A systematic review and meta‐analysis comparing complications following total joint arthroplasty for rheumatoid arthritis versus for osteoarthritis. *Arthritis & Rheumatism*, *64*(12), 3839–3849. https://doi.org/10.1002/art.37690

31. Holzapfel, D. E., Thieme, M., Kappenschneider, T., Holzapfel, S., Maderbacher, G., Weber, M., … Meyer, M. (2023). Outcome nach Gelenkersatz bei Patienten mit rheumatoider Grunderkrankung. *Zeitschrift für Rheumatologie*, *82*(10), 825–833. https://doi.org/10.1007/s00393-023-01424-4

32. Carroll, J. D., Young, J. R., Mori, B. V., Gheewala, R., Lakra, A., & DiCaprio, M. R. (2023). Total Hip and Knee Arthroplasty Surgery in the Morbidly Obese Patient: A Critical Analysis Review. *JBJS Reviews*, *11*(4). https://doi.org/10.2106/JBJS.RVW.22.00177

33. Zusmanovich, M., Kester, B. S., & Schwarzkopf, R. (2018). Postoperative Complications of Total Joint Arthroplasty in Obese Patients Stratified by BMI. *The Journal of Arthroplasty*, *33*(3), 856–864. https://doi.org/10.1016/j.arth.2017.09.067

34. AbdelSalam, H., Restrepo, C., Tarity, T. D., Sangster, W., & Parvizi, J. (2012). Predictors of Intensive Care Unit Admission After Total Joint Arthroplasty. *The Journal of Arthroplasty*, *27*(5), 720–725. https://doi.org/10.1016/j.arth.2011.09.027

**Supplementary Material:**

**Appendix 1: Medical Complications after TJA, THA and TKA**

| **Total Joint Arthroplasty (TJA)** | | | | |
| --- | --- | --- | --- | --- |
|  | **Controls**  (n=142) | **CD°IV Patients**  (n=142) | **total population**  (n=284) | **p-value** |
| **Medical Complications** n (%)  Pulmonary n (%)  Cardiological n (%)  Renal n (%)  Neurological n (%)  Gastrointestinal n (%)  Anemia n (%)  Electrolyte derangement n (%)  Urinary tract infection n (%)  Decubitus n (%)  TVT n (%) | 86 (60.6)  3 (2.1)  3 (2.1)  5 (3.5) 8 (5.6)  4 (2.8)  58 (40.8)  25 (17.6)  5 (3.5)  3 (2.1)  0 (0) | 139 (97.9)  35 (24.6)  49 (34.5)  18 (12.7)  40 (28.2)  22 (15.5)  87 (61.3)  53 (37.3)  7 (4.9)  8 (5.6)  1 (0.7) | 225 (79.2)  38 (13.4)  52 (18.3)  23 (8.1)  48 (16.9)  26 (9.2)  145 (51.1)  78 (27.5)  12 (4.2)  11 (3.9)  1 (0.4) | **<.001***  **<.001***  **<.001***  **.005***  **<.001***  **<.001***  **<.001***  **<.001***  .565*  .124*  1.0** |
| **Total Hip Arthroplasty (THA)** | | | | |
|  | **Controls**  (n=66) | **CD°IV Patients**  (n=78) | **total population**  (n=144) | **p-value** |
| **Medical Complications** n (%)  Pulmonary n (%)  Cardiological n (%)  Renal n (%)  Neurological n (%)  Gastrointestinal n (%)  Anemia n (%)  Electrolyte derangement n (%)  Urinary tract infection n (%)  Decubitus n (%)  TVT n (%) | 44 (66.7)  2 (3.0)  0 (0)  0 (0) 2 (3.0)  3 (4.5)  36 (54.5)  12 (18.2)  4 (6.1)  1 (1.5)  0 (0) | 78 (100)  19 (24.4)  27 (34.6)  10 (12.8)  23 (29.5)  14 (17.9)  59 (75.6)  28 (35.9)  3 (3.8)  4 (5.1)  0 (0) | 122 (84.7)  21 (14.6)  27 (18.8)  10 (6.9)  25 (17.4)  17 (11.8)  95 (66.0)  40 (27.8)  7 (4.9)  5 (3.5)  0 (0) | **<.001***  **<.001***  **<.001***  **.002****  **<.001***  **.013***  **.008***  **.018***  .703**  .375**  NA |
| **Total Knee Arthroplasty (TKA)** | | | | |
|  | **Controls**  (n=76) | **CD°IV Patients**  (n=64) | **total population**  (n=140) | **p-value** |
| **Medical complications** n (%)  Pulmonary n (%)  Cardiological n (%)  Renal n (%)  Neurological n (%)  Gastrointestinal n (%)  Anemia n (%)  Electrolyte derangement n (%)  Urinary tract infection n (%)  Decubitus n (%)  TVT n (%) | 42 (55.3)  1 (1.3)  3 (3.9)  5 (6.6) 6 (7.9)  1 (1.3)  22 (28.9)  13 (17.1)  1 (1.3)  2 (2.6)  0 (0) | 61 (95.3)  16 (25.0)  22 (34.4)  8 (12.5)  17 (26.6)  8 (12.5)  28 (43.8)  25 (39.1)  4 (6.3)  4 (6.3)  1 (1.6) | 103 (73.6)  17 (12.1)  25 (17.9)  13 (9.3)  23 (16.4)  9 (6.4)  50 (35.7)  38 (27.1)  5 (3.6)  6 (4.3)  1 (0.7) | **<.001***  **<.001***  **<.001***  .229*  **.003***  **.012****  .069*  **.004***  .178**  .412**  .457** |

TJA = Total joint arthroplasty, THA = Total hip arthroplasty, TKA = Total knee arthroplasty, Clavien-Dindo IV (CD°IV) = life threatening complications leading to transfer to an intermediate care unit or intensive care unit, *complications* = pooled medical and surgical complications, *medical complications* include cardiological, pulmonary, renal, thromboembolic (TVT), neurological and cerebrovascular AEs, delirium, urinary tract infection, electrolyte derangement and anemia requiring transfusion, bold values = p ≤ 0.05, *Chi-square-test. **Fishers-exact test

**Appendix 2: Multivariate Regression Analysis: Complications**

| **Total Joint Arthroplasty (TJA)** | | | | | | | |  |  |
| --- | --- | --- | --- | --- | --- | --- | --- | --- | --- |
| **Variable** | **Beta** | **SE** | **Wald** | **df** | **p-value** | **Odds Ratio** | **95% Confidence interval** |  |  |
| **Pulmonary** | 3,743 | ,685 | 29,862 | 1 | **<,001** | 42,210 | 11,02 – 161,6 |  |  |
| **Cardiological** | 4,624 | ,671 | 47,540 | 1 | **<,001** | 101,873 | 27,368 - 379,203 |  |  |
| **Renal** | 2,746 | ,614 | 19,994 | 1 | **<,001** | 15,586 | 4,676 - 51,943 |  |  |
| **Neurological** | 3,001 | ,500 | 36,006 | 1 | **<,001** | 20,105 | 7,544 -53,582 |  |  |
| **GIT** | 3,446 | ,630 | 29,928 | 1 | **<,001** | 31,368 | 9,127 - 107,803 |  |  |
| **Anemia** | ,735 | ,370 | 3,959 | 1 | **,047** | 2,086 | 1,011 - 4,305 |  |  |
| **Intercept** | -2,451 | ,345 | 50,374 | 1 | **<,001** | ,086 |  |  |  |
| **Total Hip Arthroplasty (THA)** | | | | | | | |  |  |
| **Variable** | **Beta** | **SE** | **Wald** | **df** | **p-value** | **Odds Ratio** | **95% Confidence interval** |  |  |
| **Pulmonary** | 2,447 | ,792 | 9,553 | 1 | **,002** | 11,549 | 2,448 - 54,495 |  |  |
| **Neurological** | 2,855 | ,777 | 13,514 | 1 | **<,001** | 17,367 | 3,791 - 79,562 |  |  |
| **GIT** | 2,085 | ,680 | 9,397 | 1 | **,002** | 8,046 | 2,121 - 30,518 |  |  |
| **Intercept** | -,681 | ,224 | 9,260 | 1 | **,002** | ,506 |  |  |  |
| **Total Knee Arthroplasty (TKA)** | | | | | | | |  |  |
| **Variable** | **Beta** | **SE** | **Wald** | **df** | **p-value** | **Odds Ratio** | **95% Confidence interval** |  |  |
| **Pulmonary** | 3,542 | 1,086 | 10,631 | 1 | **,001** | 34,531 | 4,108 - 290,301 |  | |
| **Cardiological** | 3,109 | ,688 | 20,420 | 1 | **<,001** | 22,405 | 5,816 - 86,302 |  | |
| **Neurological** | 1,921 | ,586 | 10,728 | 1 | **,001** | 6,827 | 2,163 - 21,550 |  | |
| **GIT** | 3,121 | 1,123 | 7,727 | 1 | **,005** | 22,658 | 2,510 - 204,537 |  | |
| **Intercept** | -1,467 | ,286 | 26,265 | 1 | **<,001** | ,231 |  |  |  |

TJA = Total joint arthroplasty, THA = Total hip arthroplasty, TKA = Total knee arthroplasty, Clavien-Dindo IV (CD°IV) = life threatening complications leading to transfer to an intermediate care unit or intensive care unit, Secondary diagnoses = pre-existing preoperative secondary diagnoses, bold values = p ≤ 0.05;

**Appendix 3: Preoperative Comorbidities**

| **Total Joint Arthroplasty (TJA)** | | | | |
| --- | --- | --- | --- | --- |
|  | **Controls**  (n=142) | **CD°IV Patients**  (n=142) | **total population**  (n=284) | **p-value** |
| **Secondary diagnoses** n (%)  Type 2 Diabetes mellitus n (%)  Preoperative anemia n (%)  Nicotine abuse n (%)  Depression n (%)  Osteoporosis n (%)  Malnutrition n (%)  Preoperative chronic CRP-Increase n (%)  Rheumatic disease n (%)  Cardiological disease n (%)  Pulmonary disease n (%)  Neurovascular disease n (%)  Haemato-oncological disease n (%)  Gastrointestinal disease n (%) | 23 (16.2)  7 (4.9)  5 (3.5)  15 (10.6)  23 (16.2)  31 (21.8)  8 (5.6)  21 (14.8)  108 (76.1)  20 (14.2)  25 (17.6)  20 (14.1)  9 (6.3) | 28 (19.7)  4 (2.8)  11 (7.7)  12 (8.5)  29 (20.4)  40 (28.2)  11 (7.7)  14 (9.9)  125 (88.0)  30 (21.1)  42 (29.6)  33 (23.2)  25 (17.6) | 51 (18.0)  11 (3.9)  16 (5.6)  27 (9.5)  52 (18.3)  71 (25.0)  19 (6.7)  35 (12.3)  233 (82.0)  50 (17.7)  67 (23.6)  53 (18.7)  34 (12.0) | .440*  .356*  .123*  .544*  .357*  .217*  .476*  .206*  **.009***  .126*  **.018***  **.048***  **.003*** |
| **Total Hip Arthroplasty (THA)** | | | | |
|  | **Controls**  (n=66) | **CD°IV Patients**  (n=78) | **total population**  (n=144) | **p-value** |
| **Secondary diagnoses** n (%)  Type 2 Diabetes mellitus n (%)  Preoperative anemia n (%)  Nicotine abuse n (%)  Depression n (%)  Osteoporosis n (%)  Malnutrition n (%)  Preoperative chronic CRP-Increase n (%)  Rheumatic disease n (%)  Cardiological disease n (%)  Pulmonary disease n (%)  Neurovascular disease n (%)  Haemato-oncological disease n (%)  Gastrointestinal disease n (%) | 12 (18.2)  2 (3.0)  2 (3.0)  7 (10.6)  16 (24.2)  12 (18.2)  4 (6.1)  7 (10.6)  50 (75.8)  8 (12.1)  10 (15.2)  9 (13.6)  3 (4.5) | 16 (20.5)  2 (2.6)  7 (9.0)  8 (10.3)  19 (24.4)  23 (29.5)  7 (9.0)  10 (12.8)  68 (87.2)  16 (20.5)  25 (32.1)  20 (25.6)  11 (14.1) | 28 (19.4)  4 (2.8)  9 (6.3)  15 (10.4)  35 (24.3)  35 (24.3)  11 (7.6)  17(11.8)  118 (81.9)  24 (16.7)  35 (24.3)  29 (20.1)  14 (9.7) | .725*  1.0**  .180**  .945*  .987*  .115*  .512*  .682*  .076*  .178*  **.018***  .073*  .054* |
| **Total Knee Arthroplasty (TKA)** | | | | |
|  | **Controls**  (n=76) | **CD°IV Patients**  (n=64) | **total population**  (n=140) | **p-value** |
| **Secondary diagnoses** n (%)  Type 2 Diabetes mellitus n (%)  Preoperative anemia n (%)  Nicotine abuse n (%)  Depression n (%)  Osteoporosis n (%)  Malnutrition n (%)  Preoperative chronic CRP-Increase n (%)  Rheumatic disease n (%)  Cardiological disease n (%)  Pulmonary disease n (%)  Neurovascular disease n (%)  Haemato-oncological disease n (%)  Gastrointestinal disease n (%) | 12 (15.8)  5 (6.6)  3 (3.9)  8 (10.5)  7 (9.2)  19 (25.0)  4 (5.3)  14 (18.4)  58 (76.3)  12 (15.8)  15 (19.7)  11 (14.5)  6 (7.9) | 12 (18.8)  2 (3.1)  4 (6.3)  4 (6.3)  10 (15.6)  17 (26.6)  4 (6.3)  4 (6.3)  57 (89.1)  14 (21.9)  17 (26.6)  13 (20.3)  14 (21.9) | 24 (17.1)  7 (5.0)  7 (5.0)  12 (8.6)  17 (12.1)  36 (25.7)  8 (5.7)  18 (12.9)  115 (82.1)  26 (18.6)  32 (22.9)  24 (17.1)  20 (14.3) | .643*  .454**  .702**  .368*  .247*  .833*  1.0**  .**032***  .050*  .356*  .338*  .361*  **.019*** |

TJA = Total joint arthroplasty, THA = Total hip arthroplasty, TKA = Total knee arthroplasty, Clavien-Dindo IV (CD°IV) = life threatening complications leading to transfer to an intermediate care unit or intensive care unit, Secondary diagnoses = pre-existing preoperative secondary diagnoses, bold values = p ≤ 0.05; *Chi-square-test. **Fishers-exact test

**Appendix 4: Potentially risk generating medication**

| **Total Joint Arthroplasty (TJA)** | | | | |
| --- | --- | --- | --- | --- |
|  | **Controls**  (n=142) | **CD°IV Patients**  (n=142) | **total population**  (n=284) | **p-value** |
| **Potentially risk generating medication**  n (%)  **Anticoagulants** n (%)  Thrombocyte aggregation inhibitors n (%)  NOAC n (%)  Coumarin-derivates n (%) | 34 (23.9)  16 (11.3)  7 (4.9)  11 (7.7) | 69 (48.6)  47 (33.1)  19 (13.4)  7 (4.9) | 103 (36.3)  63 (22.2)  26 (9.2)  18 (6.3) | **<.001***  **<.001***  **.014***  .330* |
| **RA medication**  Immunomodulatory medication n (%)  Cortisone n (%) | 4 (2.8)  6 (4.2) | 2 (1.4)  9 (6.3) | 6 (2.1)  15 (5.3) | .684**  .426* |
| **Total Hip Arthroplasty (THA)** | | | | |
|  | **Controls**  (n=66) | **CD Patients**  (n=78) | **total population**  (n=144) | **p-value**  .154* |
| **Potentially risk generating medication**  n (%)  **Anticoagulants** n (%)  Thrombocyte aggregation inhibitor n (%)  NOAK n (%)  Coumarin-derivates n (%) | 17 (25.8)  7 (10.6)  4 (6.1)  6 (9.1) | 35 (44.9)  24 (30.8)  9 (11.5)  3 (3.8) | 52 (36.1)  31 (21.5)  13 (9.0)  9 (6.3) | **.017***  **.003***  .253*  .301** |
| **RA medication**  Immunosuppressive medication n (%)  Cortisone n (%) | 1 (1.5)  4 (6.1) | 1 (1.3)  5 (6.4) | 2 (1.4)  9 (6.3) | 1.00 **  1.00** |
| **Total Knee Arthroplasties (TKA)** | | | | |
|  | **Controls**  (n=76) | **CD Patients**  (n=64) | **total population**  (n=140) | **p-value**  .154* |
| **Potentially risk generating medication**  n (%)  **Anticoagulants** n (%)  Thrombocyte aggregation inhibitor n (%)  NOAK n (%)  Coumarin-derivates n (%) | 17 (22.4)  9 (11.8)  3 (3.9)  5 (6.6) | 34 (53.1)  23 (35.9)  10 (15.6)  4 (6.3) | 51 (36.4)  32 (22.9)  13 (9.3)  9 (6.4) | **<.001***  **<.001***  **.018***  1.0** |
| **RA medication**  Immunosuppressive medication n (%)  Cortisone n (%) | 3 (3.9)  2 (2.6) | 1 (1.6)  4 (6.3) | 4 (2.9)  6 (4.3) | .625 **  .412** |

TJA = Total joint arthroplasty, THA = Total hip arthroplasty, TKA = Total knee arthroplasty, Clavien-Dindo IV (CD°IV) = life threatening complications leading to transfer to an intermediate care unit or intensive care unit, Secondary diagnoses = pre-existing preoperative secondary diagnoses, Potentially risk generating medication = medications that have already been taken preoperatively and cause potential risk factors, anticoagulants include pooled thrombocyte aggregation inhibitors, new oral anticoagulants and coumarin-derivates, NOAC = new oral anticoagulants, RA medication = medication for rheumatic diseases, immunomodulatory medication includes disease-modifying antirheumatic drugs (DMARDs) like Methotrexat, biologicals, Sulfasalazin, Leflunomid, Hydroxychloroquin and Azathriopin, bold values = p ≤ 0.05; *Chi-square-test. **Fishers-exact test

**Appendix 5: Multivariate Regression Analysis: preoperative secondary diseases and medication**

| **Total Joint Arthroplasty (TJA)** | | | | | | | |  |
| --- | --- | --- | --- | --- | --- | --- | --- | --- |
| **Variable** | **Beta** | **SE** | **Wald** | **df** | **p-value** | **Odds Ratio** | **95% Confidence interval** |  |
| **Cardiological** | ,703 | ,356 | 3,888 | 1 | **,049** | 2,020 | 1,004 - 4,062 |  |
| **GIT** | 1,101 | ,427 | 6,652 | 1 | **,010** | 3,008 | 1,303 - 6,944 |  |
| **HFRS** | ,128 | ,041 | 9,987 | 1 | **,002** | 1,137 | 1,050 - 1,230 |  |
| **Anticoagulants** | ,989 | ,270 | 13,365  5 | 1 | **<,001** | 2,688 | 1,582 - 4,567 |  |
| **Intercept** | -1,525 | ,373 | 16,717  7 | 1 | **<,001** | ,218 |  |  |
| **Total Hip Arthroplasty (THA)** | | | | | | | |  |
| **Variable** | **Beta** | **SE** | **Wald** | **df** | **p-value** | **Odds Ratio** | **95% Confidence interval** |  |
| **HFRS** | ,108 | ,048 | 5,088 | 1 | **,024** | 1,115 | 1,014 – 1,225 |  |
| **Anticoagulants** | ,836 | ,368 | 5,152 | 1 | **,023** | 2,308 | 1,121 - 4,751 |  |
| **Intercept** | -,561 | ,281 | 3,987 | 1 | **,046** | ,571 |  |  |
| **Total Knee Arthroplasty (TKA)** | | | | | | | |  |
| **Variable** | **Beta** | **SE** | **Wald** | **df** | **p-value** | **Odds Ratio** | **95% Confidence interval** |  |
| **GIT** | 1,250 | ,547 | 5,223 | 1 | **,022** | 3,489 | 1,195 - 10,190 |  |
| **Anticoagulants** | 1,401 | ,381 | 13,535 | 1 | **<,001** | 4,057 | 1,924 - 8,556 |  |
| **Intercept** | -,863 | ,244 | 12,467 | 1 | **<,001** | ,422 |  |  |

TJA = Total joint arthroplasty, THA = Total hip arthroplasty, TKA = Total knee arthroplasty, Clavien-Dindo IV (CD°IV) = life threatening complications leading to transfer to an intermediate care unit or intensive care unit, Secondary diagnoses = pre-existing preoperative secondary diagnoses, bold values = p ≤ 0.05;
